# Large-scale genomic phylogeography provides insights into evolutionary history and conservation priorities of the white-bellied pangolin (*Phataginus tricuspis*)

**DOI:** 10.1093/molbev/msag049

**Published:** 2026-02-26

**Authors:** Tong Tong Gu, Tian Ya Zhai, Yu Jiang, Bao Tong Qi, Feng Yang, Zhong Xu Zhang, Rui Yu, Oladipo Omotosho, Olajumoke Morenikeji, Hua Rong Zhang, Jing Yang Hu, Li Yu

**Affiliations:** School of Life Sciences, State Key Laboratory for Conservation and Utilization of Bio-Resource in Yunnan, Yunnan University, Kunming, China; School of Life Sciences, Yunnan Normal University, Kunming, China; School of Life Sciences, State Key Laboratory for Conservation and Utilization of Bio-Resource in Yunnan, Yunnan University, Kunming, China; School of Life Sciences, State Key Laboratory for Conservation and Utilization of Bio-Resource in Yunnan, Yunnan University, Kunming, China; School of Life Sciences, State Key Laboratory for Conservation and Utilization of Bio-Resource in Yunnan, Yunnan University, Kunming, China; Kadoorie Farm and Botanic Garden, Lam Kam Road, Tai Po, Hong Kong SAR, China; Forest Police Corps of Yunnan Public Security Department, Kunming, China; Kunming Natural History Museum of Zoology, Kunming Institute of Zoology, Chinese Academy of Sciences, Kunming, China; Department of Veterinary Medicine, University of Ibadan, Ibadan, Nigeria; Department of Veterinary Medicine, University of Ibadan, Ibadan, Nigeria; Kadoorie Farm and Botanic Garden, Lam Kam Road, Tai Po, Hong Kong SAR, China; School of Life Sciences, State Key Laboratory for Conservation and Utilization of Bio-Resource in Yunnan, Yunnan University, Kunming, China; School of Life Sciences, State Key Laboratory for Conservation and Utilization of Bio-Resource in Yunnan, Yunnan University, Kunming, China; Southwest United Graduate School, Kunming, China

**Keywords:** white-bellied pangolin, genome resequencing, evolutionary history, conservation genetics, African biodiversity

## Abstract

The white-bellied pangolin (*Phataginus tricuspis*) serves as a critical biogeographic indicator for understanding faunal diversification in African rainforests and is a priority for conservation due to its status as the most heavily trafficked and endangered mammal. However, the species’ evolutionary history and the genetic consequences of population decline remain unclear. In this study, we conducted comprehensive phylogeographic and conservation genomic studies of 209 whole genomes, including 100 newly sequenced genomes, and 215 mitogenomes covering all geographic ranges. Our findings reveal four whole-genome genetic lineages and six mitochondrial genetic lineages, uncovering mito-nuclear discordance driven by deep mitochondrial divergence and the replacement of some mitochondrial lineages by nuclear lineages. We suggest that Pleistocene refugia and river barriers are hypothesized to have contributed to the pattern of genetic differentiation and biogeographic diversification. Demographic history reconstruction indicates that, historically, the population size dynamics were likely correlated with glacial-interglacial cycles. However, the recent sharp decline in population size can be attributed to overexploitation driven by international trade. The genetic consequence analyses and evolutionary potential simulation reveal that the Nigeria and West Africa lineages exhibit lower levels of genetic diversity, higher levels of inbreeding and genetic load, and lower survival status and future evolutionary potential, than the other lineages, indicating the need for urgent attention and priority conservation action. Our results provide novel insights into the evolutionary history and conservation priorities for white-bellied pangolins and offer a valuable phylogeographic and conservation framework for guiding conservation efforts to safeguard African rainforest biodiversity.

## Introduction

The white-bellied pangolin (*Phataginus tricuspis*), also known as the African common pangolin or tree pangolin, has a widespread sub-Saharan distribution in African tropical rainforest, ranging from western to central Africa with eastern and southern limits reaching southwestern Kenya and northern Angola, respectively (Waterman et al. 2014; Gaubert et al. 2016). Two main hypotheses have been proposed to explain faunal diversification in African rainforests (Nicolas et al. 2010): the Pleistocene forest refuge hypothesis, which suggests that forest fragmentation during Pleistocene glacial maxima promoted isolation and subsequent diversification of forest-associated taxa; and the river barrier hypothesis, which posits that major tropical rivers restrict population/species dispersal and drive diversification by acting as geographical barriers. Due to their limited dispersal capabilities and heavy reliance on large trees, white-bellied pangolins serve as valuable biogeographic indicators for understanding faunal diversification in African rainforests (Gaubert 2011; Gaubert et al. 2016). Their distribution patterns provide key insights for testing the influence of the Pleistocene forest refuge and river barrier hypotheses on the evolutionary history of rainforest species (Quérouil et al. 2003; Anthony et al. 2007; Huhndorf et al. 2007; Nicolas et al. 2008). Meanwhile, overexploitation driven by local demand for bushmeat and traditional medicine (Ingram et al. 2018), and international trafficking of their scales have led to severe population declines, making them the most heavily trafficked and endangered mammals (Challender et al. 2014, 2020; Heinrich et al. 2017; Ingram et al. 2018; Tinsman et al. 2023). Understanding the species’ evolutionary history and the genomic consequences associated with population decline is essential for its conservation management and global action planning.

The evolutionary history of white-bellied pangolin has been studied for over a decade using both molecular and morphological data (Kingdon and Hoffmann 2013; Gaubert et al. 2016; Gaubert et al. 2018; Ferreira-Cardoso et al. 2020; Tinsman et al. 2023; Din Dipita et al. 2024). Nevertheless, debate persists regarding the number of genetic lineages, with estimates varying from three to six across different markers. Combined analyses of mitochondrial (mtDNA) and nuclear genes (Gaubert et al. 2016), and mitogenome (Gaubert et al. 2018) have identified six divergent genetic lineages (referred to as mtDNA lineages): the Western Africa lineage (WAfr), the Ghana lineage (Gha), the Dahomey Gap lineage (DG), the Western Central Africa lineage (WCA), the Gabon lineage (Gab), and the Central Africa lineage (CA). Based on divergence times of the six lineages, the Pleistocene refuge hypothesis, rather than the river barrier hypothesis appears to explain the observed biogeographic distribution pattern (Gaubert et al. 2016). Nevertheless, analysis based on only nuclear genes recovered just three lineages/lineage groups compared to the six based on the mtDNA: a Western Africa lineage group (WAfr, Gha, and DG), the WCA lineage, and a Central Africa lineage group (CA and Gab) (Gaubert et al. 2016). Subsequent morphological analyses, however, supported the DG lineage from the Western Africa lineage group being separate as it has a distinct skull shape; this resulted in four lineages, unlike the nuclear genes analysis (Ferreira-Cardoso et al. 2020). Recently, low-coverage depth genomic data (average coverage < 5-fold) analyses proposed the occurrence of five lineages, including the four lineages identified by skull morphology (Ferreira-Cardoso et al. 2020) and an additional lineage (Cameroon_Manyu lineage), which is separated from the WCA lineage (Tinsman et al. 2023). Therefore, the genetic lineage divergence controversy remains unresolved, and the phylogeographic history needed to be confirmed, along with determining which hypothesis adequately explains the population diversification of white-bellied pangolin.

In addition, research investigating the genetic consequences of population decline and evolutionary history of white-bellied pangolin is limited. Numerous studies have used mtDNA, nuclear genes, and microsatellites to attempt species identification and illegal trade origin tracing (Hsieh et al. 2011; Gaubert et al. 2016; Mwale et al. 2017; Aguillon et al. 2020; Zhang et al. 2020; Zanvo et al. 2022; Gossé et al. 2023; Bernathova et al. 2024; Din Dipita et al. 2024; Yeo et al. 2024), and a few studies addressing the genetic consequences are based on only one individual or lineage (Damas et al. 2022; Gu et al. 2023; Heighton et al. 2023; Houck et al. 2023). A systematic investigation of all white-bellied pangolin lineages is lacking.

Here, we conducted a large-scale phylogeographic and conservation genomics analysis of 209 whole genomes, 100 of which were newly generated, and 215 mitogenomes covering all geographic populations. Our study resolves longstanding controversies regarding white-bellied pangolin genetic lineages based on the nuclear genes and morphological evidence, uncovering the reasons for underlying mito-nuclear discordances. We discuss how different hypotheses can explain the biogeographic pattern and evolution history, and we elucidate the genetic consequences, survival status, and future evolutionary potential of different genetic lineages associated with population reduction. Our findings provide critical insights into the evolutionary history and conservation priorities for white-bellied pangolins, establishing a phylogeographic and conservation framework for understanding and protecting African rainforest biodiversity.

## Results

### Whole-genome dataset and mitochondrial dataset

**Figure 1:**
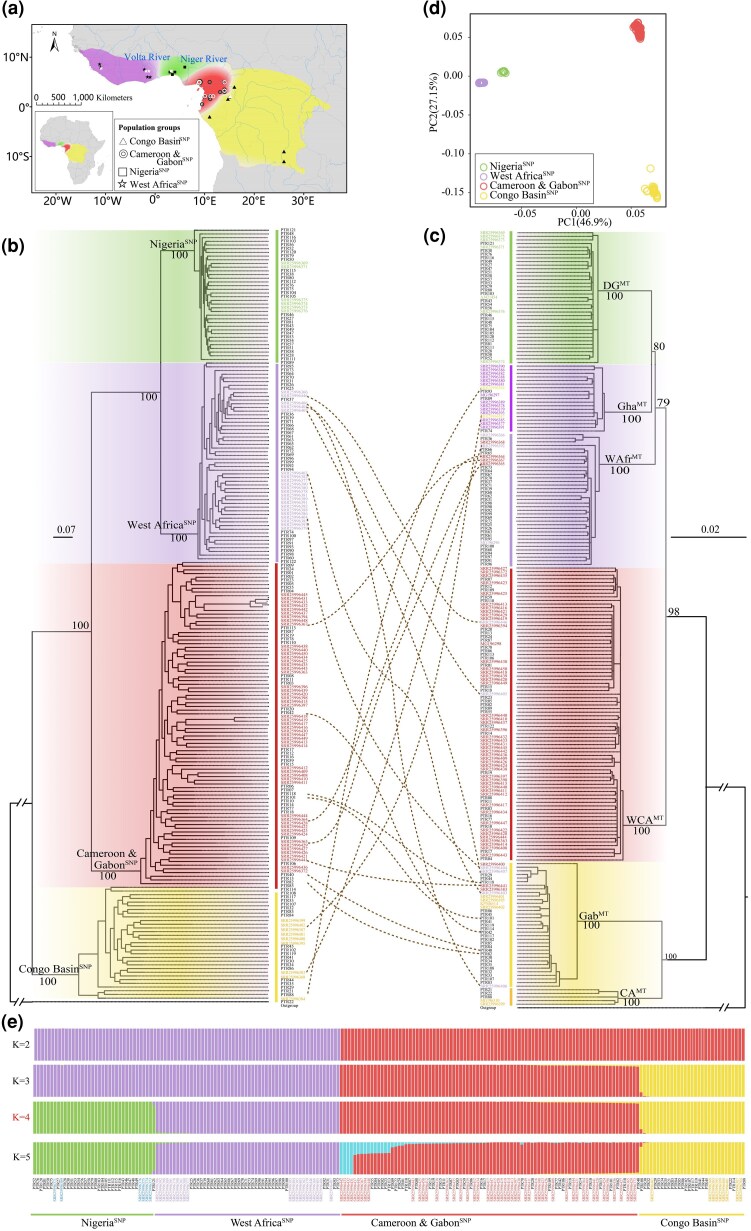
Sample information and genetic landscape. a) The distribution range of white-bellied pangolin obtained from the IUCN. The different colors on the map represent the four whole-genome lineages determined in this study. The 26 origin locations from Tinsman et al. (2023) are indicated by black symbols and relate to 87 individuals. The 11 locations indicated in white represent the results from the 122 samples from confiscated material subsequently assigned to these areas. The different symbols represent the different whole-genome lineages. b) Phylogenetic tree inferred from autosome SNPs (ML); c) Phylogenetic tree inferred from mitochondrial sequences (ML); d) Principal component analysis (PCA); e) Admixture analysis. The optimal *K* value was four (Figure S2). The bootstrap value for each lineage shown under the branches of the phylogenic tree, the samples colored consistently for the genetic lineages represent the samples with detailed geographic origins from published data and the black samples represent the 122 confiscated individuals with assigned locations (b–c).

We obtained whole-genome data from 209 white-bellied pangolins (Fig. 1a and Tables S1 and S2), including 100 newly generated individuals with an average 45.50-fold coverage (ranging from 30.69 to 54.11) and average mapping rate of 96.34% (ranging from71.91% to 99.91%) (Table S1). The data also include 22 published individuals with high coverage (over 30-fold) (Damas et al. 2022; Gu et al. 2023; Houck et al. 2023), and 87 published individuals with lower coverage depth (average coverage was about 5.04-fold) (Tinsman et al. 2023) (Table S2). The single-nucleotide polymorphism (SNP) calling and filtering produced a total of 5,352,077 bp and 29,489,280 bp high-quality autosomal SNP datasets of all 209 individuals and 122 high coverage individuals, respectively. In addition, a mitochondrial dataset from 215 individuals comprising 209 newly assembled and six previously published (Gaubert et al. 2018) individuals was also produced.

### Population structures and admixtures

Based on the autosome SNP dataset of 209 individuals, we explored the population structures and admixtures of white-bellied pangolins. Phylogenetic analysis strongly supported the four whole-genome genetic lineages that were associated with the distinct and nonoverlapping geographic regions (with robust node supports = 100%) (Fig. 1b). The Congo Basin^SNP^ diverged first (including samples from the Republic of Congo, Central African Republic, Democratic Republic of Congo, and eastern Cameroon), followed by the Cameroon and Gabon^SNP^ (western and southern Cameroon, Gabon, and Equatorial Guinea), and the sister grouping between the West Africa^SNP^ lineage (Ghana and Sierra Leone) and the Nigeria^SNP^ lineage (Nigeria). However, the mitochondrial phylogeny analysis based on 215 individuals recovered all six mitochondrial genetic lineages (with robust node supports ≥ 70%) that have been widely reported using mitochondrial sequences (Fig. 1c and Figure S1) (Gaubert et al. 2018; Zhang et al. 2020; Yeo et al. 2024). The sister cluster of the CA^MT^ (eastern Cameroon, Central African Republic, and Democratic Republic of Congo) and Gab^MT^ (eastern and southern Cameroon, Gabon, Equatorial Guinea, Central African Republic, and Republic of Congo) lineages diverged first, followed by the WCA^MT^ (western and southern Cameroon, Gabon, and Equatorial Guinea) lineage and the WAfr^MT^ lineage (Sierra Leone and Ghana), respectively. The sister group of the Gha^MT^ (Sierra Leone and Ghana) and DG^MT^ (Nigeria) lineages was the last to diverge. We found the WCA^MT^ lineage consistent with the Cameroon and Gabon^SNP^ lineage and the DG^MT^ lineage corresponded to the Nigeria^SNP^ lineage, but both the CA^MT^ and Gab^MT^ lineages, and the WAfr^MT^ and Gha^MT^ lineages were, respectively, integrated into the Congo Basin^SNP^ lineage and the West Africa^SNP^ lineage.

In addition to the difference in the number of lineages, some individuals also have inconsistent genetic lineages on both trees. Six individuals were classified into the West Africa^SNP^ lineage, with two individuals placed in the WCA^MT^ lineage and four individuals in the Gab^MT^ lineage; nine individuals were classified into the Cameroon and Gabon^SNP^ lineage, with four individuals placed in the WAfr^MT^ lineage and five individuals in the Gab^MT^ lineage; three individuals were clustered into the Congo Basin^SNP^ lineage, with two individuals placed in the Gha^MT^ lineage and one individual in the WAfr^MT^ lineage (Fig. 1b, c). However, these types of patterns were not observed in the Nigeria^SNP^ lineage (Fig. 1b, c)

Principal component analyses (PCA) (Patterson et al. 2006) and admixture analyses (Alexander et al. 2009) also revealed four whole-genome genetic lineages which were consistent with the phylogenetic analysis using the SNP dataset (Fig. 1d, e). The first PCA axis (PC1) successfully separated all white-bellied pangolin into two lineage groups (a West Africa^SNP^ and Nigeria^SNP^ lineage group and a Cameroon and Gabon^SNP^ and Congo Basin^SNP^ lineage group), then the second PCA axis (PC2) further separated each lineage group into two genetic lineages (Tracy-Widom, *P* < 0.001) (Fig. 1d). The admixture analyses showed the lowest cross-validation error when *K* = 4 (Figure S2) and supported four genetic lineages in white-bellied pangolin. The two lineage groups distinguished by the first PC were also supported when *K* = 2. In addition, some individuals exhibited an admixture signal within the two lineage groups when *K* = 3 to 5, wherein several individuals showed incongruent phylogenetic placements between the whole-genome and mitochondrial analyses. The *F_ST_* values of each pair among four genetic lineages were ranged from 0.376 (between Cameroon and Gabon^SNP^ and Congo Basin^SNP^ lineages) to 0.672 (between Nigeria^SNP^ and Congo Basin^SNP^ lineages) (Table S3).

### Divergence time estimation

**Figure 2:**
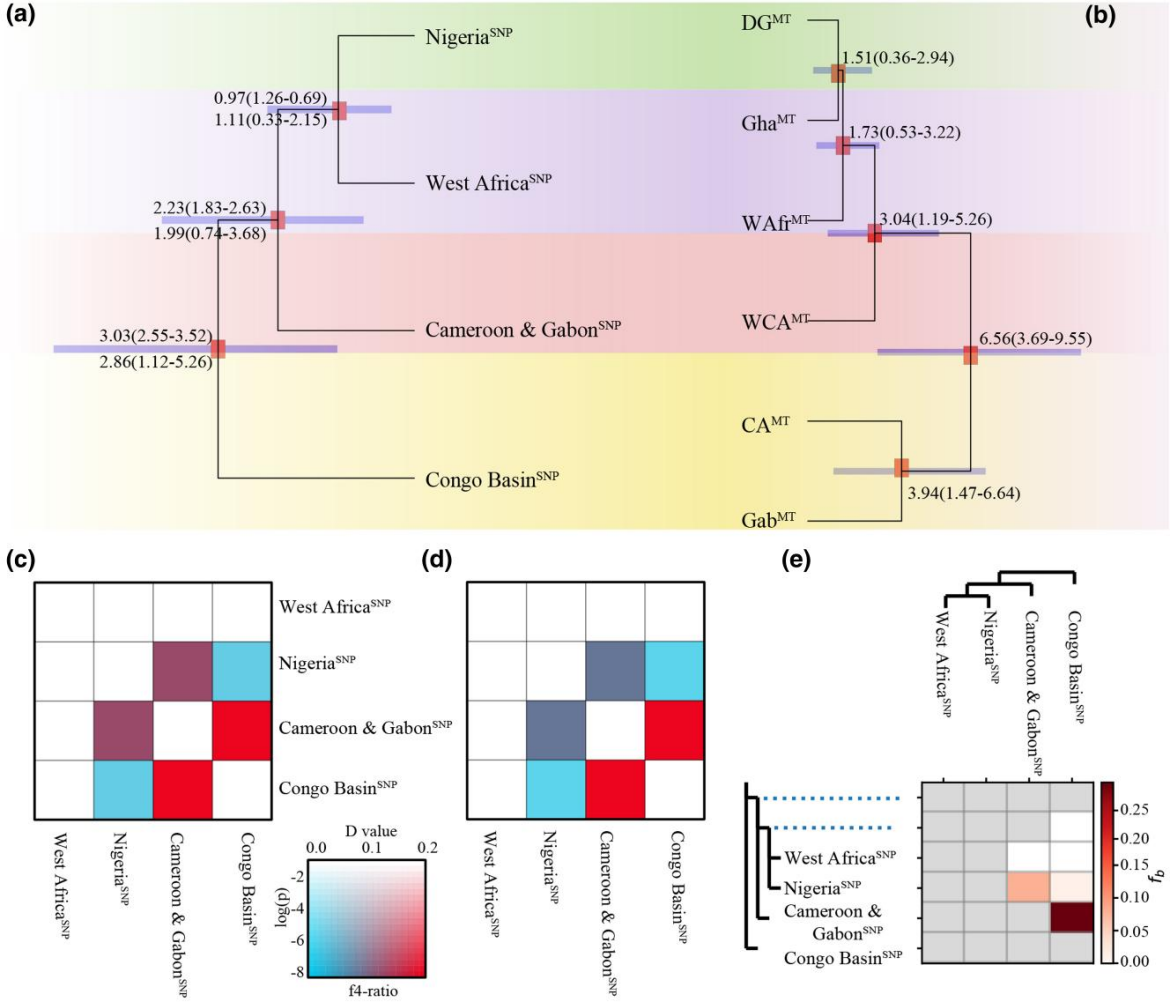
Divergence time and gene flow. a) Divergence time inferred from genome-wide SNP data using MCMCTREE and fastsimcoal2; b) Divergence time inferred from mitochondrial sequences. Interspecific divergence times are shown on the branches, including median values and their 95% HPDs. The red boxes on the nodes indicate full bootstrap values at the nodes representing relationships within white-bellied pangolin. c–e) The results of gene flow analyses using the D statistic (c), f4-ratio test (d), and the f-branch method (e).

The divergence time estimation using two genome-wide methods produced consistent results (Fig. 2a, Figure S3 and Table S4). Analyses with fastsimcoal2 v.2.7 (Excoffier et al. 2013) indicated that the divergence time of the four whole-genome genetic lineages of white-bellied pangolin ranging from 3.03 (95% HPD: 3.52 to 2.55) to 0.97 (95% HPD: 1.26 to 0.69) million years ago (mya) (Fig. 2a and Figure S3). The divergence time using MCMCTREE (Yang 2006) based on fossil correction points showed that all the TMRCA estimates for the four whole-genome genetic lineages and most of their internal nodes ranged from 2.86 (95% HPD: 5.26 to 1.12) to 1.11 (95% HPD: 2.15 to 0.33) mya (Fig. 2a and Table S4). Comparatively, divergence times based on mitochondrial data showed that the TMRCA estimates for the six mitochondrial lineages and their internal nodes spanned the range 6.56 (95% HPD: 9.55 to 3.69) to 1.51 (95% HPD: 2.94 to 0.36) mya (Fig. 2b and Table S5), ie earlier than the divergence time estimates based on genome-wide data.

### Gene flow

Analyses of the genome-wide SNP data based on the D statistic, the f4-ratio test, and the f-branch method (Fig. 2c to e and Table S6) revealed the strongest gene flow between the Congo Basin^SNP^ and Cameroon and Gabon^SNP^ lineages (*D* = 0.25, |Zscore|>5, *P* < 0.001), followed by lower gene flow between the Cameroon & Gabon^SNP^ and Nigeria^SNP^ lineages (*D* = 0.13, |Zscore|>5, *P* < 0.001), and the lowest gene flow between the Congo Basin^SNP^ and Nigeria^SNP^ lineages (*D* = 0.02, |Zscore|>3, *P* < 0.001). We did not detect gene flow signals between Congo Basin^SNP^ and West Africa^SNP^, or between Cameroon and Gabon^SNP^ and West Africa^SNP^.

### Demographic history

**Figure 3:**
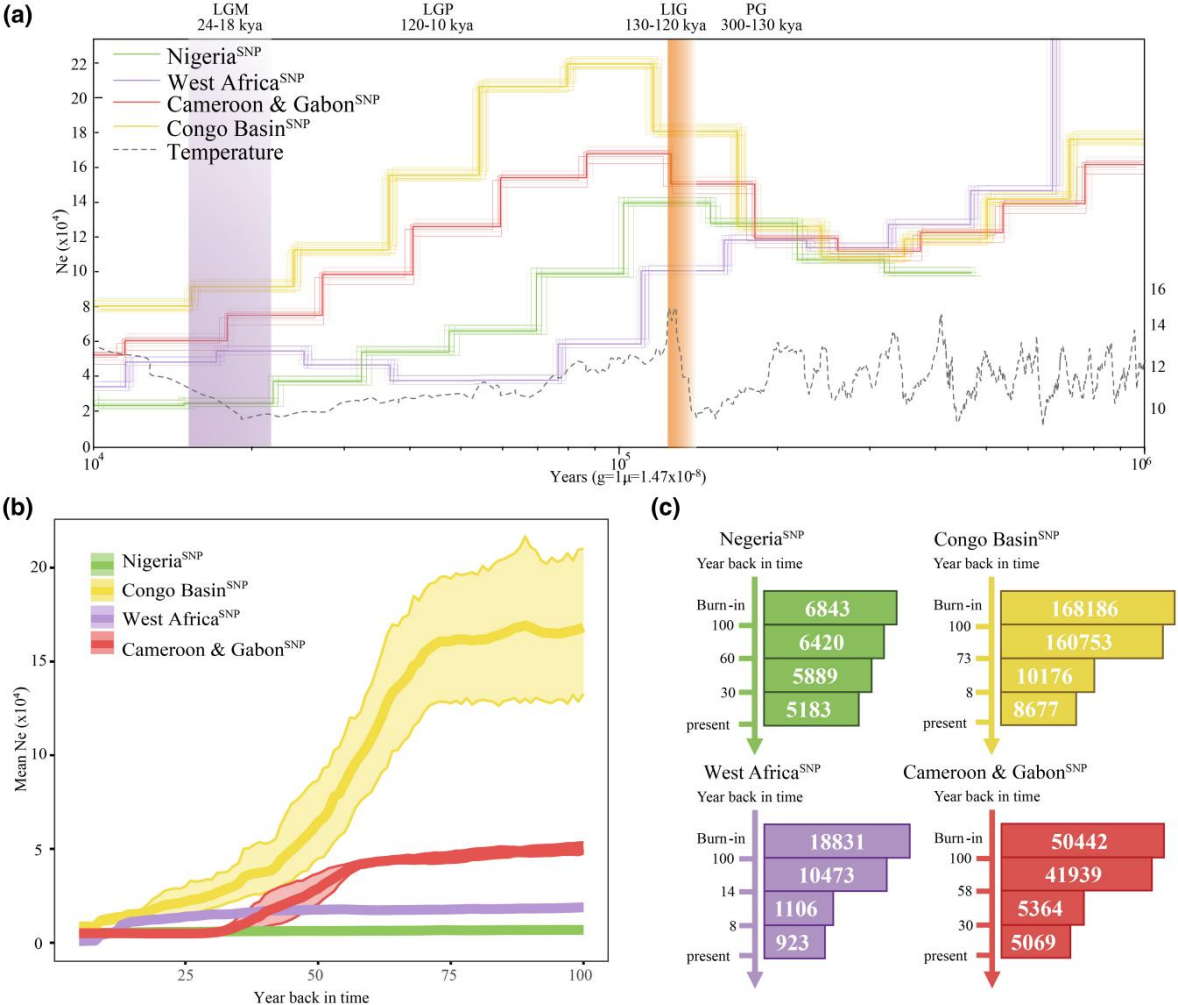
Demographic history. a) Demographic history analyze based on MSMC2. b–c) Temporal *Ne* over recent time periods reconstructed using GONE.

Demographic history analysis indicated that population bottlenecks and expansions were consistent with climatic changes (cold/warm cycles) (Fig. 3a). Three of the four lineages, i.e. Nigeria^SNP^, Cameroon and Gabon^SNP^, and Congo Basin^SNP^, experienced a similar history of population dynamics in the late Pleistocene, with consistent population bottlenecks and population expansions during the penultimate glacial (PG, 0.30-0.13 mya) and the Last Interglacial Period (LIG, 0.13 to 0.12 mya), respectively, and then continuous declines in effective population size during the last glacial period (LGP, 0.12 to 0.01 mya), reaching a minimum during the last glacial maximum (LGM, 0.024 to 0.018 mya). It is worth noting that the West Africa^SNP^ lineage showed a different demographic history, undergoing population decline from the LIG to the LGP, followed by an expansion that peaked during the LGM.

The recent population history reconstruction using GONE software revealed that, in the past century, three of the four genetic lineages of white-bellied pangolin have experienced a sharp population decline (Fig. 3b, c) (Santiago et al. 2020). The Congo Basin^SNP^ and Cameroon and Gabon^SNP^ lineages, which have larger historical population sizes, experienced an abrupt population decline 73 and 58 years ago, respectively, with the effective population size decreasing by approximately 94.6% (declining from 160,753 to 8,677 individuals) and 87.9% (declining from 41,939 to 5,069 individuals). The West Africa^SNP^ lineage, with its smaller historical population size, has experienced a recent population decline, lasting until 14 years ago, decreasing approximately 91.2% (declining from 10,473 to 923 individuals). In comparison to the abrupt population decline in these three lineages, Nigeria^SNP^ has maintained a smaller population size and a slower decline rate, with its effective population size decreasing by approximately 19.3% over the past century (from 6,420 to 5,183 individuals).

### Genetic consequence

**Figure 4:**
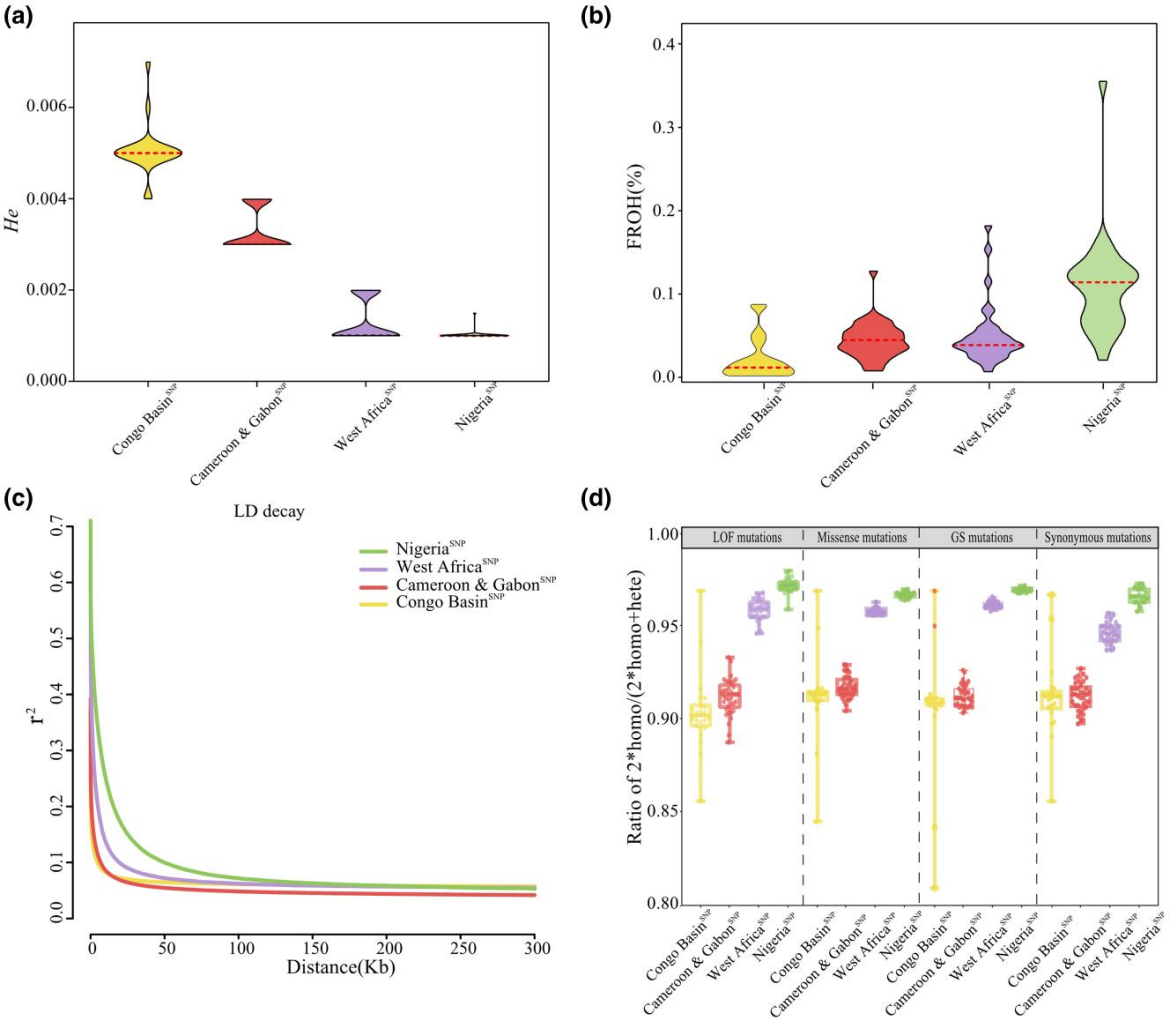
Genetic diversity, inbreeding levels, linkage disequilibrium and genetic load evaluation. a) Whole-genome genetic diversity based on the average heterozygosity (*He*) among all individuals for each genetic lineage; b) Inbreeding levels calculated based on the average inbreeding coefficient (*F*_ROH_) among all individuals for each genetic lineage; c) Linkage disequilibrium (LD) for each genetic lineage; d) Evaluations of genetic load based on LOF mutations, missense mutations, deleterious-GS mutations, and synonymous mutations among all individuals for each genetic lineage.

We conducted genetic consequence analyses which include evaluating the genetic diversity, inbreeding level, linkage disequilibrium (LD), and genetic load. Low genetic diversity is typically associated with a high level of inbreeding, low LD decay, and serious genetic load. We found that the genetic diversity of these four lineages ranged from 0.0011 to 0.0049 with an average of 0.0027, as follows: Congo Basin^SNP^ lineage (average = 0.0049, 0.004 to 0.007) > Cameroon and Gabon^SNP^ lineage (average = 0.0034, 0.0028 to 0.0038) > West Africa^SNP^ lineage (average = 0.0015, 0.0014 to 0.0016) > Nigeria^SNP^ lineage (average = 0.0011, 0.0005 to 0.0013) (*P* < 0.01, Wilcoxon rank-sum test; Fig. 4a). Conversely, reduced genetic diversity is frequently associated with increased inbreeding levels (Sanchez-Barreiro et al. 2021; De Ferran et al. 2022). The genomic inbreeding coefficients (*F*_ROH_) of the four lineages ranged from 0.020% to 0.107% with an average of 0.054%, as follows: Nigeria^SNP^ lineage (average = 0.107%, 0.019 to 0.355%) > West Africa^SNP^ lineage (average = 0.048%, 0.005% to 0.181%) > Cameroon & Gabon^SNP^ lineage (average = 0.043%, 0.006% to 0.126%) > Congo Basin^SNP^ lineage (average = 0.020%, 0 to 0.086%) (*P* < 0.01, Wilcoxon rank-sum test; Fig. 4b). In addition, genome-wide LD analyses of LD decay with a reduced *r*^2^ correlation coefficient demonstrated that Nigeria^SNP^ (100 kb) > West Africa^SNP^ (70 kb) > Cameroon and Gabon^SNP^ (50 kb) > Congo Basin^SNP^(30 kb) (Fig. 4c).

Increasing inbreeding levels can lead to an elevated genetic load and support the homozygosity of recessive deleterious alleles, which potentially compromise gene functionality and diminishes individual fitness (Charlesworth and Willis 2009; Hu et al. 2020b). Consistent with the inbreeding analysis, the ratio of homozygous to homozygous and heterozygous sites followed the order: Nigeria^SNP^ lineage (0.971 for loss-of-function [LOF]; 0.966 for missense mutations; 0.969 for the Grantham score [GS]; 0.965 for synonymous mutations) > West Africa^SNP^ lineage (0.957 for LOF; 0.957 for missense mutations; 0.961 for the GS; 0.945 for synonymous mutations) > Cameroon & Gabon^SNP^ lineage (0.910 for LOF; 0.916 for missense mutations; 0.911 for the GS; 0.912 for synonymous mutations) > Congo Basin^SNP^ lineage (0.902 for LOF; 0.911 for missense mutations; 0.904 for the GS; 0.910 for synonymous mutations) (*P* < 0.01, Wilcoxon rank-sum test, Fig. 4d). All of the genetic consequence analyses indicated that, among the four genetic lineages, the Nigeria^SNP^ and West Africa^SNP^ lineages had lower genetic diversity, higher levels of inbreeding, faster LD decay, and more severe genetic load.

### Survival status and evolutionary potential simulation

**Figure 5:**
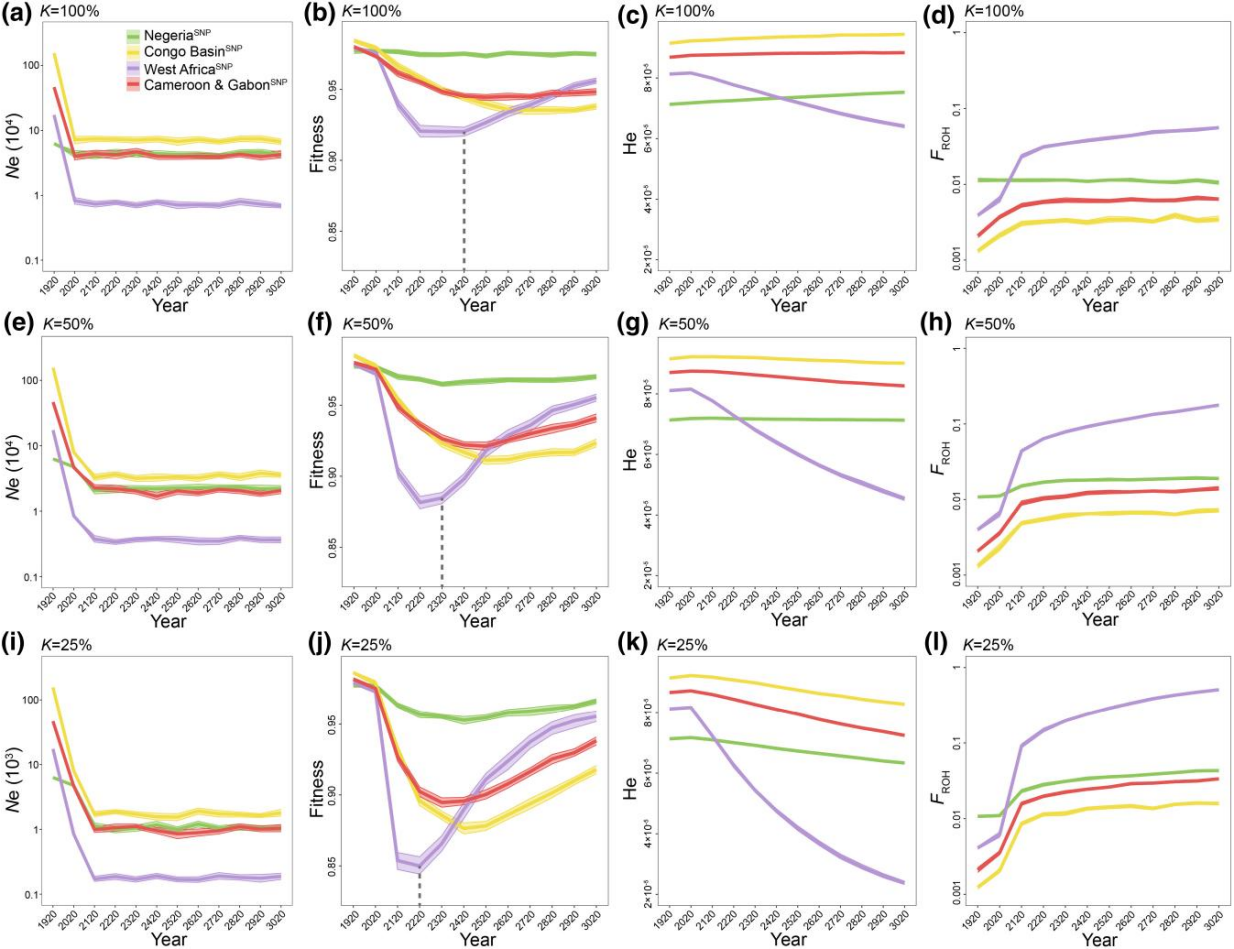
Simulation of evolutionary potential over the next 1,000 years. a to d) The effective population size (a), fitness (b), heterozygosity (c), and inbreeding level (d) over the next 1,000 years when *K* is equal to 100% of the current *N*e. e to h) The effective population size (e), fitness (f), heterozygosity (g), and inbreeding level (h) over the next 1,000 years when *K* is equal to 50% of the current *N*e. i to l) The effective population size (i), fitness (j), heterozygosity (k), and inbreeding level (l) over the next 1,000 years when *K* is equal to 25% of the current *N*e. The gray dashed lines represent the time when significant recovery of fitness is predicted to occur in the West Africa^SNP^ population.

We first assessed the future survival status and evolutionary potential of these four genetic lineages over the next 1,000 years (from 2,020 to 3,020), assuming the carrying capacity (*K*) of the current effective population size remaining stable (*K* = 100%) (Fig. 5a to f). We developed effective population size (*Ne*) models for each genetic lineage of white-bellied pangolin over the past century (from 1920 to 2020) (Fig. 3b, c). Simulations showed that the *Ne* would remain stable without further decline, suggesting that, in the absence of human intervention, none of the four genetic lineages will go extinct in the future (Fig. 5a). Both the Congo Basin^SNP^ and Cameroon and Gabon^SNP^ lineages have experienced significant declines in fitness (Wilcoxon rank-sum test, *P* < 0.001), but, in the simulation, the declines would be halting 2,620 and 2,420, respectively (Fig. 5b). However, both lineages are predicted to maintain a high level of heterozygosity and a low level of inbreeding (Fig. 5c, d). The West Africa^SNP^ lineage will suffer a significant fitness decline due to a pronounced *Ne* reduction (Wilcoxon rank-sum test, *P* < 0.001), but unlike the Congo Basin^SNP^ and Cameroon & Gabon^SNP^ lineages, the fitness will recover significantly around 2,420 (Wilcoxon rank-sum test, *P* < 0.001) (Fig. 5b). Conversely, the heterozygosity will decrease, and the inbreeding levels will increase significantly (Wilcoxon rank-sum test, *P* < 0.001) (Fig. 5c, d). For the Nigeria^SNP^ lineage, the fitness is expected to remain stable in the future (Fig. 5b), while the heterozygosity and inbreeding level show a continuous increase (Fig. 5c) and a continuous reduction in the future (Fig. 5d) (Wilcoxon rank-sum test, *P* < 0.001), respectively. However, both the Nigeria^SNP^ lineage and the West Africa^SNP^ lineage, which respectively experienced long term small population size and recent population decline, exhibit reduced survival status and lower evolutionary potential compared with the larger populations of the Congo Basin^SNP^ and Cameroon and Gabon^SNP^ lineages (Fig. 5c, d).

Considering that white-bellied pangolins continue to face intense poaching pressure, both for local consumption and illegal trade as well as habitat degradation and fragmentation driven by infrastructure development such as new ports, roads, and railways (Hughes et al. 2020; Yang et al. 2021; Tinsman et al. 2023; Assou et al. 2025; Emogor et al. 2025), we simulated the future survival status and evolutionary potential of the four genetic lineages in the next 1,000 years when the carrying capacity decreases (*K* = 50% and *K* = 25%, ie at 50% or 25% of current *Ne*) to reflect the species’ ongoing demographic pressures of population decline (Fig. 5e to l). Interestingly, all four genetic lineages would maintain smaller population sizes and none of them would face extinction based on replicate simulations and 95% confidence (Fig. 5e and i). The fitness of all four lineages showed a trend of initial decrease and subsequent recovery (Fig. 5f, j), suggesting that the smaller population size of the four genetic lineages would have the ability to clear deleterious mutations and recover fitness. This smaller population size would have a higher capacity for this especially in the West Africa^SNP^ lineage, for which the predicted time for significant recovery of fitness would accelerate from 2,420 to 2,320 and 2,220, for *K* = 50% and *K* = 25%, respectively (Fig. 5b, f, and j). However, further population decline would result in a significant reduction in heterozygosity and an increasing level of inbreeding, significantly reducing the evolutionary potential across all four genetic lineages (Wilcoxon rank-sum test, *P* < 0.001) (Fig. 5g, h, k, and l). When *K* = 50%, heterozygosity in the Nigeria^SNP^, Congo Basin^SNP^, Cameroon and Gabon^SNP^ and West Africa^SNP^ lineages would decline by 0.83%, 2.38%, 5.60%, and 44.36%, respectively (Fig. 5g, h). Concurrently, inbreeding level would increase by 70.54%, 200.83%, 289.04%, and 2,568.67%, respectively (Fig. 5g, h). When *K* = 25%, heterozygosity in the same lineages is predicted to decline by 11.70%, 10.18%, 16.95%, and 70.87%, respectively, while inbreeding level would increase by 293.64%, 664.42%, 838.55%, and 8,030.99%, respectively (Fig. 5k, l). These results indicated that the West Africa^SNP^ lineage would exhibit the poorest survival status and the lowest evolutionary potential compared to the other genetic lineages, followed by the Nigeria^SNP^ lineage (Fig. 5g, h, k, and l).

## Discussion

In this study, we conducted a large-scale phylogeographic and conservation genomics analysis of white-bellied pangolins. We identified four whole-genome genetic lineages based on phylogeny, PCA and admixture analyses (Fig. 1b, d, and e). These comprised the three genetic lineages previously identified by nuclear gene analysis (Gaubert et al. 2016) with the Nigeria^SNP^ lineage (referred to as the DG lineage in a previous study) as an independent genetic lineage due to this lineage located in Dahomey Gap. This region has long been recognized as a major biogeographic divide separating the Upper and Lower Guinean forest blocks (Moreau 1969), while also serving as a potential refuge for ecologically tolerant species (Booth 1958). Climatic fluctuations over time resulted in alternating periods of habitat connectivity and fragmentation, which likely limited gene flow across the region. This complex history of isolation and reconnection contributed to the pronounced genetic divergence observed between the Nigerian and the Cameroon and Gabon lineages in the analysis, notably in PCA. However, we did not find support for the Cameroon_Manyu lineage, which was identified by Tinsman et al. (2023) as a distinct lineage. Instead, we suggest that this group belongs to the Cameroon and Gabon^SNP^ genetic lineage. These four whole-genome genetic lineages are also consistent with morphological analyses of skull shape (Ferreira-Cardoso et al. 2020), thus resolving longstanding debates regarding the number of genetic lineages of white-bellied pangolins when considering nuclear genes (Gaubert et al. 2016; Tinsman et al. 2023) and morphology (Ferreira-Cardoso et al. 2020). In contrast to the whole-genomic dataset, the mitochondrial dataset recovered all six previously reported mitochondrial lineages (Gaubert et al. 2016, 2018; Zhang et al. 2020; Yeo et al. 2024) (Fig. 1c). When we excluded the DG^MT^ lineage, the other five mitochondrial lineages were not associated with clear geographical boundaries and we found more than two mitochondrial genetic components in the same distribution area (Figure S1), indicating historical hybridization events. We speculate that the mito-nuclear discordance found in white-bellied pangolin genetic lineages is due to a deep mitochondrial divergence, revealed by the absence of nuclear-genomic differentiation between two lineages, but significant mitochondrial differentiation (Zhang et al. 2019). The nuclear genome of the Gab^MT^ lineage may have completely replaced the CA^MT^ lineage and finally formed the Congo Basin^SNP^ lineage during the period 2.86 to 3.03 mya. In addition, the nuclear genome of the WAfr^MT^ lineage completely replaced the Gha^MT^ lineage during the early Pleistocene (0.97 to 1.11 mya) and formed the West Africa^SNP^ whole-genomic lineage (Fig. 2a). These results provide evidence for the divergent evolutionary histories of the nuclear and mitochondrial genomes in the white-bellied pangolin.

The divergence times of the four genomic genetic lineages (2.86 to 3.03, 1.99 to 2.23, and 0.97 to 1.11 mya, Fig. 2a) coincide with shifts in marine sediment sequences and the onset/intensification of high-latitude glacial cycles during the Pliocene-Pleistocene (deMenocal 1995, 2004). This temporal correspondence supports the hypothesis that the African climate periodically oscillated between markedly wetter and drier conditions around 2.8, 1.8, and 1.0 mya, respectively (deMenocal 1995, 2004). The Pleistocene refugia hypothesis, which suggests the presence of fragmented habitat caused by extreme drought associated with climate change, could explain the genetic lineage diversification and the evolutionary history of the highly forest-dependent and widely distributed white-bellied pangolin. Compared with previous fossil data (deMenocal 2004), the white-bellied pangolin, often referred to as a “living fossil,” strongly supports the suggestion that Pliocene-Pleistocene climate change was a key driver of biological diversification in the African rainforest. In addition to the Pleistocene refugia hypothesis, there has been discussion about the effect of the Dahomey Gap, which is found between the Volta and Niger rivers (Gaubert et al. 2016). Our results indicate that the Niger river acted as a biogeographic barrier separating the Cameroon and Gabon^SNP^ lineage from the sister group of Nigeria^SNP^ and West Africa^SNP^ lineages, and that the Volta river further divided this sister group. These results indicate that the Volta and Niger rivers acted as natural barriers, thereby providing support for the river barrier hypothesis (Quérouil et al. 2003; Anthony et al. 2007; Nicolas et al. 2011). Thus, the rivers contributed to the differentiation of genetic lineages of white-bellied pangolin. Overall, our results not only validate the Pleistocene refugia hypothesis, but also support the river barrier hypothesis in explaining the biogeographic pattern and evolution history of the white-bellied pangolin.

We found that the Nigeria^SNP^, Cameroon and Gabon^SNP^, and Congo Basin^SNP^ lineages exhibited population bottlenecks and expansion consistent with the glacial-interglacial cycles. These fluctuations were probably related to the reduction in the extend of suitable habitats during cold, dry glacial periods, and subsequent recovery during warmer, wetter interglacial periods (Fig. 3a). Similar patterns have been documented in sympatric species, such as gorillas and chimpanzees (Xue et al. 2015), African leopards (Paijmans et al. 2021), and red river hogs (Xie et al. 2022a), as well as neighboring-sympatric species like African elephants (Palkopoulou et al. 2018), lions (De Manuel et al. 2020), rhinoceroses (Liu et al. 2021), baboons (Rogers et al. 2019; Sørensen et al. 2023), and warthogs (Garcia-Erill et al. 2022; Xie et al. 2022a). In contrast, the West Africa^SNP^ lineage showed a unique demographic history, undergoing population expansion during the cold, dry climate of the LGM (Fig. 3a). This pattern may be attributed to regional climatic influences. Previous studies suggest that the upwelling of cold deep-sea water in the Upper Guinea Block may have moderated the dry and cold LGM climate in this region, promoting the persistence of rainforest habitats through increased cloud cover, reduced solar radiation, and frequent drizzle and mist (Leal 2004). These favorable ecological conditions likely facilitated the expansion of the West African lineage.

Once abundant in Africa, the white-bellied pangolin has been the most harvested and illegally traded species (Tinsman et al. 2023). Our demographic reconstruction detected significant population declines over the past 100 years (Fig. 3b, c). Among the four genetic lineages, the Congo Basin^SNP^ lineage had the largest historic population size and suffered the most serious population shrinkage (Fig. 3b, c), which is consistent with the most serious illegal trade activity: an estimated increase from 0.42 to 2.71 million animals hunted in Central Africa over the past four decades (Ingram et al. 2018). In addition, the Cameroon and Gabon^SNP^ lineage showed a 87.9% decline post-1967 (Fig. 3b, c), aligning with seizure data identifying southern Cameroon, Equatorial Guinea, and Gabon as the poaching hotspots between 2012 and 2018 (Tinsman et al. 2023). Furthermore, the West Africa^SNP^ lineage experienced rapid decline nearly a decade ago (∼91.2%) (Fig. 3b, c), coinciding with the shift from demand for local bushmeat to the growing international illegal trade during the last 15 years (Challender 2012; Ingram et al. 2019; Gossé et al. 2023). Interestingly, Nigeria^SNP^ has maintained a smaller population size with a slower rate of decline over the past century (Fig. 3b, c). This may be due to white-bellied pangolin in this region only being used for local trade (Zanvo et al. 2022). Indeed, few samples from this lineage were found in the international illegal trade (Zhang et al. 2019; Tinsman et al. 2023; Yeo et al. 2024). This pattern, although seemingly at odds with Nigeria's status as a major pangolin scale export hub (Zhang et al. 2020), can be explained by its primary role as a transit point for scales sourced from neighboring countries rather than a significant country of origin. This is consistent with previous findings that most scales trafficked in Nigeria come from elsewhere (Tinsman et al. 2023), which accounts for the relatively limited impact on local populations. Our results underscore the necessity of allocating efforts and resources to enhance law enforcement and population monitoring, and to curtail illegal hunting and the illegal wildlife trade in the poaching hotspots relevant to the other three genetic lineages (Zhang et al. 2020; Tinsman et al. 2023).

Both historical climate-driven population fluctuations and contemporary anthropogenic pressures have significantly shaped the genetic structure of the white-bellied pangolin. We found a more than 5-fold variation in the genetic diversity among lineages (Fig. 4a). Notably, the Congo Basin^SNP^ (0.0049) and Cameroon and Gabon^SNP^ (0.0034) lineages had the highest genetic diversity among all known pangolin species, including the ninth pangolin species identified on the basis of genetic evidence (Gu et al. 2023; Heighton et al. 2023). In contrast, the Nigeria^SNP^ (0.0011) and West Africa^SNP^ (0.0015) lineages showed significantly lower genetic diversity, comparable to critically endangered species like the Chinese pangolin (0.0013) and Malayan pangolin from southeast Asian (0.0014) (Hu et al. 2020b), and endangered Indian pangolin (0.0010) and Giant pangolin (0.0018) (Gu et al. 2023). The lower genetic diversity, faster LD decay, higher inbreeding levels, and more serious genetic load (Fig. 4b to d), suggest that the Nigeria^SNP^ and West Africa^SNP^ lineages may have less evolutionary potential, stronger inbreeding depression and a diminished capacity to adapt to environmental changes.

Although there is no risk of extinction of the four lineages (Fig. 5a, e, and i) under different carrying capacities (*K* = 100%, 50%, and 25%, respectively), their future survival status and evolutionary potential is quite different. The Congo Basin^SNP^ and Cameroon and Gabon^SNP^ lineages had similar effective population sizes as the Nigeria^SNP^ lineage (Fig. 4c, d), but the severe population decline over the past century will lead to a decline in their future fitness, although gradually their fitness, genetic diversity and inbreeding level will become stable (Fig. 5b to d). In comparison, the Nigeria^SNP^ lineage with its small population reduction was predicted to have relatively stable future fitness and increased future evolutionary potential (Fig. 5b to d). Notably, the recent rapid and drastic population reduction resulted in the West Africa^SNP^ lineage having the smallest population (Fig. 4c, d), leading to a rapid decline in its predicted future fitness (Fig. 5b). However, a small population can quickly eliminate harmful mutations (Khan et al. 2021; Kleinman-Ruiz et al. 2022; Xie et al. 2022b), so the fitness will also quickly recover (Fig. 5b), although the future evolutionary potential of this small population will continue to decline due to limited gene flow (Fig. 5c, d).

In addition, the fitness recovery time of the small population advances, indicating the increasing ability to eliminate harmful mutations with a decreasing carrying capacity and further population size decline (Fig. 5b, f, and j). However, the genetic diversity will continue to decrease and inbreeding level will increase in the future (Fig. 5g, h, k, and d), especially in the West Africa^SNP^ lineage. This comes at the cost of significant loss of evolutionary potential, which may impair the West Africa^SNP^ lineage's ability to withstand future environmental changes, highlighting the need for immediate intervention and indicating that this lineage should be prioritized in future conservation efforts. Addressing future conservation challenges not only requires reducing habitat loss and illegal activities, but also implementing genetic rescue strategies such as translocation or assisted gene flow to enhance genetic diversity and offset the effects of inbreeding. Our study is the first to evaluate the conservation status and future prospects of the four genetic lineages of white-bellied pangolin, and suggests that the West Africa^SNP^ and Nigeria^SNP^ lineages require urgent attention and conservation action should be prioritized over other lineages.

## Conclusion

Our study resolves longstanding controversies regarding white-bellied pangolin genetic lineages with respect to nuclear genetic and morphological evidence. We have uncovered the underlying causes of mito-nuclear discordances, proposed hypotheses to explain the biogeographic patterns and evolution history, and elucidated the genetic consequences, survival status, and future evolutionary potential of different genetic lineages associated with population reduction. Our findings provide critical insights into the evolutionary history and conservation priorities for white-bellied pangolins. Additionally, they establish a phylogeographic and conservation framework that can be applied to understand and protect African rainforest biodiversity.

## Materials and methods

### Sample collection and sequencing

We collected a total of 100 confiscated white-bellied pangolins scale samples from Yunnan, China (samples donated by Yunnan Provincial Forest Public Security Bureau, and the Animal Branch of the Germplasm Bank of Wild Species of Chinese Academy of Sciences) and Hong Kong, China (samples donated by the Agriculture, Fisheries and Conservation Department of the Hong Kong SAR Government to Kadoorie Farm and Botanic Garden) (Table S1). The pieces of cellular tissue attached to the scales were used for DNA extraction. The outer surface (0.5 mm) of tissues was first removed using a surgical blade to eliminate possible surface contaminants. Then, a small section of tissue was excised, transferred to a 2-ml Eppendorf tube and washed with ethanol and then 10X Phosphate buffered saline. DNA from these samples was subsequently extracted using a Magen Hipure DNA Micro Kit (Magen, China). All necessary research permits and ethical approvals were obtained (No: YNUCARE20210003).

Illumina sequencing libraries with 500-bp inserts were generated for the samples from Yunnan, China, and sequenced on the Illumina NovaSeq platform at Berry Genomics Co. (Beijing, China) to generate 150-bp paired-end reads. The samples from Hong Kong, China were used to construct proprietary DNBSEQ sequencing libraries with 300 to 500-bp inserts, and sequenced on the BGISEQ-500 sequencing technology platform at BGI Hong Kong Co. (Hong Kong, China) to generate 150-bp paired-end reads. All the paired-end reads (raw data) were trimmed to remove adapter sequences and low-quality sequences to obtain clean data. For Illumina data, all reads containing >20% low-quality nucleotides (Q ≤ 5) or >10% ambiguous nucleotides were removed. For BGISEQ data, all reads containing more than 50% low-quality bases (Q ≤ 20) or more than 3% ambiguous nucleotides were removed.

Besides the genomic sequences newly generated here (NCBI BioProject ID: PRJNA1129867), the 109 whole-genome resequencing data for white-bellied pangolins were downloaded from NCBI (Damas et al. 2022; Gu et al. 2023; Houck et al. 2023; Tinsman et al. 2023), and one Giant pangolin (*Smutsia gigantea*) was chosen as an outgroup for the analysis (Gu et al. 2023) (Table S2).

### Variant calling and filtering

We used BWA-MEM (Li and Durbin 2009) to align each high-quality resequencing dataset to the chromosome assembly level genome of white-bellied pangolin (Houck et al. 2023). BAM alignment files were generated using SAMtools v.1.3 (Li et al. 2009). PCR duplicates were removed using PICARD (http://picard.sourceforge.net). The Genome Analysis Toolkit (GATK) v.4.1.8 (McKenna et al. 2010) was used for SNP calling. Indel (insertion–deletion) realignment was performed using the IndelRealigner algorithm. A gvcf file for each sample was obtained using the “HaplotypeCaller” module, and all gvcf files were merged using “CombineGVCFs” in GATK. SNP calling was then conducted with “GenotypeGVCFs” and “SelectVariants” was used to obtain candidate SNPs. To generate high-quality SNPs, the candidate SNPs were filtered using GATK with the following criteria: QUAL < 30.0 || QD < 2.0 || MQ < 40.0 || FS > 60.0 || SOR > 3.0 || MQRankSum < −12.5 || ReadPosRankSum < −8.0 || SB≥−1.0. Then, SNPs with missing data over 25%, minor allele frequency below 5% and depth below three, as well as indels and nonbiallelic SNPs were filtered using VCFtools v.0.1.13 (Danecek et al. 2011). We used Kinship-based Inference for Genome-wide association studies (KING) (Manichaikul et al. 2010) to remove samples representing duplicate individuals. Kinship coefficients were estimated with the “−make-king-table” command in PLINK v.2.0 (Purcell et al. 2007), the output of which reflects the proportion of SNPs with identical states (IBS0, identity by state zero) between individuals. Negative coefficients indicate no relationship between individuals, while positive coefficients indicate genealogical links. No duplicate individuals were found in our dataset. For the autosome SNP dataset, the candidate sex-chromosomes from the assembled genomes identified by Houck et al. (2023) were excluded.

### Mitochondrial dataset generation

We adopted two strategies to assemble mitochondrial genes for all individuals. First, mitochondrial assembly was performed using the whole-genome resequencing data from all individuals. The published mitochondrial genes of white-bellied pangolin (MG196310) were used as the reference for the mitochondrial assembly (Gaubert et al. 2018). The paired-end reads from the genomic resequencing data for each individual were first combined into a single fastq file, and then assembled with default parameters in MITObim v.1.9.1 (Hahn et al. 2013), which was run 3-5 times independently. However, some of the individuals could not be assembled successfully due to the lower coverage, so we determined the mitochondrial sequences for these individuals according to consensus sequences between the aligned short reads and the corresponding published mitochondrial genes (MG196310) using BWA-ALN (Hu et al. 2020b). In addition, six published mitochondrial genes representing six divergent geographic lineages were downloaded from NCBI and added to the mitochondrial analyses (Gaubert et al. 2018). All mitochondrial sequences were used in the analyses after excluding the highly repetitive and poorly assembled *D-loop* region. Sequences were aligned using PRANK v.170427 (Loytynoja 2014) and ambiguous sites were removed using Trimal v.1.4.1 (gap = all) (Capella-Gutierrez et al. 2009).

### Assigning confiscated samples to their geographic origins

We assignment the geographical origin of 122 unknown samples, including 100 individuals newly obtained in this study and 22 published individuals (Damas et al. 2022; Gu et al. 2023; Houck et al. 2023), using the R package *OriGen* (Rañola et al. 2014) with the method presented by Tinsman et al. (2023). The *OriGen* model divides the region of each white-bellied pangolin range into pixels and generates allele frequency surfaces for each SNP, and then applies Bayes’ rule to calculate the posterior probabilities to localize the origin of a given individual. The pixel with the highest posterior probability was considered to be the predicted geographic origin (latitude and longitude) of an individual. Moreover, the highly differentiated 96 SNPs selected from distinct geographic lineages (genetic lineages) have been confirmed to be effective in assigning confiscated white-bellied pangolins to precise latitude and longitude coordinates within 500 kilometers (Tinsman et al. 2023). We extracted these 96 SNPs for each individual and successfully determined the geographical origin of 122 unknown samples (Fig. 1a, Table S2). Of these, 33 individuals were from Sierra Leone and Ghana, 30 from Nigeria, 22 from Cameroon's southern border with The Republic of the Congo, and 37 from Southern, Eastern, Southeastern, and Western Cameroon (Fig. 1a, Table S2).

### Phylogenetic analyses, population admixture and principal component analysis

Phylogenetic analyses were performed based on autosomal SNPs and mitochondrial sequences. The autosomal SNPs were thinned by VCFtools v.0.1.13 (Danecek et al. 2011) based on randomly extracting a site from every 10-kb length window size to avoid the influence of linkage disequilibrium (LD) between loci; 190,000 bp SNPs were obtained after thinning. Autosomal SNPs were used to construct a Maximum Likelihood (ML) tree using RaxML v.8.2.12 (Stamatakis 2014) based on the GTRGAMMA model with 1,000 bootstraps. The input files used python script vcf2phylip v.2.8 (Ortiz 2019) to convert VCF files to PLYLIP files. The mitochondrial sequences (3,964 bp) were also used to reconstruct the ML gene-tree as described above. The dataset of autosomal SNPs was obtained to conduct admixture and principal component analysis (PCA) using Admixture v.1.2.3 (Alexander et al. 2009) and the smartPCA program from the Eigensoft v.4.2 package (Patterson et al. 2006), respectively. Numbers of ancestral clusters (*K*) from one to ten were considered and the optimal *K* value was determined using cross-validation (Alexander et al. 2009). The PCA plots were drawn based on the two principal components PC1 and PC2 with the largest contribution and the Tracy-Widom test was conducted. The Fst values between each pair of genetic lineages were estimated by VCFtools v.0.1.13 (Danecek et al. 2011) based on a 50-kb window size.

### Divergence times estimation

Divergence time estimation was based on three datasets: autosomal SNPs, connected single-copy orthologous coding sequences, and mitochondrial sequences. For autosomal SNPs, fastsimcoal2 v.2.7 (Excoffier et al. 2013) was used to calculate the divergence time with the optimal population history model. EasySFS (Coffman et al. 2016) was used to obtain unfolded joint SFS from different populations. We set the mutation rate per generation to 1.47 × 10^−8^ (Choo et al. 2016) and the generation time to one year (Zhang et al. 2016). We used 500,000 coalescent simulations and 50 optimization (ECM) cycles to run each model, each with a bidirectional average gene flow between populations. To avoid local maxima, we ran 100 repetitions independently and calculated the 95% confidence intervals. We compared the fit of two models on the basis of Akaike information criterion (AIC) values (Akaike 1974) and considered the model with the smallest AIC value to be optimal (Figure S3). The results suggested the model in which the West Africa^SNP^ population diverged earlier than the Nigeria^SNP^ population was the most suitable.

A single-copy orthologous coding sequence dataset was generated, including giant pangolin (PRJNA414857) (Heighton et al. 2023), Chinese pangolin (PRJNA529513) (Hu et al. 2020b), white-bellied pangolin (Houck et al. 2023), and two outgroups from domestic dog (PRJNA68156) (Lindblad-Toh et al. 2005) and domestic cat (PRJNA773801) (Buckley et al. 2020). Single-copy orthologous sequences were extracted using OrthoFinder v.2.5.4 (Emms and Kelly 2015). For the remaining white-bellied pangolin lineages without de novo genome assemblies, we selected one individual with a high coverage depth (∼30-fold) from each lineage, respectively, to extract the corresponding gvcf files, and remerged them using “GenotypeGVCFs” in GATK v.4.1.8 (McKenna et al. 2010). Finally, we obtained the 1,951 single-copy orthologous sequences, aligned them using PRANK v.170427 (Loytynoja 2014), and removed ambiguous sites using Trimal v.1.4.1 (gap = all) (Capella-Gutierrez et al. 2009).

The mitochondrial dataset included outgroups from dog (GeneBank accession AB499817) and cat (KP202278), and published data from giant pangolin SRR25256620 and SRR25461945) (Gu et al. 2023), Chinese pangolin (SRR9018600) (Hu et al. 2020b), black-bellied pangolin (SRR25256536) (Gu et al. 2023), and white-bellied pangolins with high-quality data assembled in this study representing each lineage.

The divergence times for the single-copy orthologous coding sequences and mitochondrial sequence datasets were estimated using MCMCTREE, PAML v.4.9 (Yang 2006) with the GTR model. Calibration points were based on the fossil records as follows: between cat and dog at 37.3 to 66 million years ago (mya) (Fox et al. 2010; Benton et al. 2015), between pangolin and Carnivora at 66 to 87 mya (Fox et al. 2010; Gaubert et al. 2018), and the crown pangolins at 31 to 45 mya (DL Gebo and Rasmussen 1985; Gaudin et al. 2009).The molecular divergence time between white-bellied pangolin and black-bellied pangolin at 5.9 to 12.8 mya was also used in mitochondrial analyses (DL Gebo and Rasmussen 1985; Gaudin et al. 2009). Birth (*λ*), death (*μ*), and sampling (*ρ*) priors of *λ*=1, *μ*=1, and *ρ*=0 were used. The transition/transversion rate ratio (kappa gamma), the shape parameter for rate heterogeneity between sites (alpha gamma), and the prior on rates (rgene gamma) were specified as (6, 2), (1, 1), and (1, 12.91), respectively. Burn-in was set at 200,000 iterations, and sampling was performed every 50 iterations until 2,000,000 samples had been gathered.

### Gene introgression analysis

We used the Dinvestigate program in Dsuite (Malinsky et al. 2021) to evaluate the gene flow between different lineages, as revealed in the phylogeny analysis within white-bellied pangolin using autosomal SNPs from 122 individuals. Three approaches—D statistics (Durand et al. 2011), the f4-ratio (Patterson et al. 2012), and the f-branch method (Malinsky et al. 2018)—were used for this purpose. Giant pangolin was selected as the outgroup. For the D statistics and f4-ratio analyses, we ran “Dsuite Dtrios” by inputting the merged vcf file, the tree file, and the sample sets file. For f-branch analysis, we performed “Dsuite Fbranch” with the phylogenetic tree and used the output of the “Dsuite Dtrios” analysis to map the gene flow intensity to the phylogenetic tree topology.

### Evaluation of genetic diversity, inbreeding level, and LD analyses

We assessed genetic diversity by calculating the average heterozygosity (*He*) among all individuals for each lineage. The heterozygosity for each individual was calculated as the ratio of the number of heterozygous sites to the total number of callable sites across the genome. The inbreeding level was calculated according to the average inbreeding coefficient (*F*_ROH_) among all individuals for each lineage. Long runs of homozygosity (ROH) were identified by PLINK v.2.0 (Purcell et al. 2007) using the following parameter settings: -homozyg -homozyg-window-snp 20 -homozyg-kb 100. The inbreeding coefficient (*F*_ROH_) for each individual was calculated as the total length of runs of homozygosity divided by the total length of the autosomes covered by SNPs (L_Auto_, as described by McQuillan et al. (2008)). Longer ROHs indicate more recent inbreeding. LD analysis was performed for each lineage of white-bellied pangolin using PopLDdecay v.3.40 (Zhang et al. 2019). We plotted the decay curve of the *r*-squared statistic (*r*^2^), which is the correlation coefficient between two focal loci of interest.

### Deleterious mutation patterns

Deleterious mutations (ie genetic load) are considered to disrupt gene function and are thus expected to substantially reduce the mean fitness of each individual (Mattila et al. 2012). We used SnpEff v.4.3t (Cingolani et al. 2012) to evaluate the genetic load level by categorizing the derived allele mutations in the coding regions of each individual into LOF, missense, and synonymous mutations. The genotypes of dominant alleles in all individuals (ie alleles in which more than 50% of individuals are homozygous) and the homozygous alleles that are also found in the outgroup were defined as ancestral genotypes (Feng et al. 2019). We built databases from the annotations and reference genome sequences of the white-bellied pangolin (Houck et al. 2023). Input files in VCF format were used to annotate SNPs and to assign mutation categories to the input SNPs for each individual. LOF mutations included premature stop codons (nonsense) and splice site disrupting single-nucleotide variants (SNVs). The deleterious load was estimated as the ratio of the number of derived homozygous sites (two per site) to both the derived homozygous and heterozygous sites (two per homozygous site and one per heterozygous site) for each category (Robinson et al. 2016; Feng et al. 2019). The deleteriousness of missense mutations was also evaluated using the GS (Grantham 1974), a measure of the physical/chemical consequences of amino acid changes. Grantham scores equal to or greater than 150 were considered deleterious (Li et al. 1984).

### Demographic history reconstruction

We performed demographic history analysis with a coalescent approach that allows up to eight diploid individual genomes to be analyzed (MSMC2, https://github.com/stschiff/msmc2) (Schiffels and Durbin 2014). The distribution of the time to the most recent common ancestor (TMRCA) between two alleles across all chromosomes was estimated by assuming a mutation rate per generation of 1.47 × 10^−8^ (Choo et al. 2016), an average generation time of 1 year (Zhang et al. 2015, 2016), and 100 bootstrap replicates.

In addition, we employed GONE (Santiago et al. 2020), a program designed to estimate recent effective population size based on LD, to estimate the recent *Ne* of various lineages. GONE has been shown not to be affected by natural selection (Novo et al. 2022), and retains accuracy even for small sample sizes (Santiago et al. 2020). Pairs of sites within 0.004 cM (parameter hc was set to 0.004) were used to reduce bias arising from recent population substructure (Santiago et al. 2020). The recombination rate (parameter cMMb) was set to 1 cM Mb^−1^ (typical among mammals), generation time was set to one year, and other parameters used default values. For each population, we randomly sampled 50,000 SNPs from each chromosome to estimate LD, performing 40 bootstrapping iterations and calculating geometric mean values each time. The analysis was repeated 100 times for each population, and 95% confidence intervals were calculated. Finally, effective population size from 100 recent generations was estimated.

### Genomic simulation of the future evolutionary potential

We used SLiM v.3.6 (Haller and Messer 2019) to simulate the future evolutionary potential (including fitness, heterozygosity and inbreeding coefficient) for each lineage of white-bellied pangolin under a non-Wright–Fisher model. Based on the recent demographic history simulated by GONE (Fig. 4b), we set the carrying capacity (*K*) to 100%, 50% and 25% of the current models to run forward-in-time simulations for the next 1,000 years. We simulated a diploid genome with 56 chromosomes (Houck et al. 2023) and 20,000 genes, with the length for each gene set to 1,500 bp, representing the total exon length of the protein-coding gene. The mutation rate was set at 1.47 × 10^–8^ per site per generation (Choo et al. 2016) and the generation time was one year (Zhang et al. 2016). The recombination rate between genes was 1 × 10^–3^ per site per generation to reach an effective recombination rate of 1 × 10^–8^ within a region of 100 kb. The ratio of deleterious to neutral mutations was set to 2.31:1 (Huber et al. 2017). The dominance coefficient (*h*) and selection coefficient (*s*) for deleterious alleles were obtained on the basis of the fitness effects distribution for human data (Kim et al. 2017). Considering that the more deleterious the alleles, the stronger their recessive nature, we used the mixed dominance coefficient (*h* = 0 when *s*<–0.01 and *h* = 0.25 when *s*≥−0.01) (Kyriazis et al. 2021; Xie et al. 2022b). Random natural disasters are modeled in future populations by adding random deaths and the probability of death for each generation is derived from the beta distribution with *α*=0.5 and *β*=8. We assumed that it was a hermaphrodite random mating population, with each generation of individuals over one year of age reproducing. We ran 25 replicates for each model and output results every 100 generations. We used the results for all replicates by calculating average values and 95% confidence intervals. All scripts used in the evolutionary potential simulations have been made publicly available at: https://github.com/Zhaitianya428/white-bellied-pangolin.

## Supplementary Material

msag049_Supplementary_Data

## Data Availability

The resequencing short-read Fastq files generated in this study have been deposited in the NCBI (https://www.ncbi.nlm.nih.gov/) archive under BioProject PRJNA1129867.
