# Supplementary material for: Large-scale genomic phylogeography provides insights into evolutionary history and conservation priorities of the white-bellied pangolin (Phataginus tricuspis)
Source: Mol Biol Evol. 2026 Feb 26;43(3):msag049. doi: 10.1093/molbev/msag049 (PMC12978532; doi:10.1093/molbev/msag049)
Supplement: msag049_Supplementary_Data [file msag049_supplementary_data.pdf]

Supplementary Information for

Large-scale genomic phylogeography provides insights into evolutionary history and conservation priorities of the white-bellied pangolin (*Phataginus tricuspis*)

Tong Tong Gu[1,2,*], Tian Ya Zhai[1,*], Yu Jiang[1,*], Bao Tong Qi[1], Feng Yang[3], Zhong Xu Zhang[4], Rui Yu[5], Oladipo Omotosho[6], Olajumoke Morenikeji[6], Hua Rong Zhang[3,#], Jing Yang Hu[1,#], Li Yu[1,7,#]

[1] School of Life Sciences, State Key Laboratory for Conservation and Utilization of Bio-Resource in Yunnan, Yunnan University, Kunming, China

[2] School of Life Sciences, Yunnan Normal University, Kunming, China

[3] Kadoorie Farm and Botanic Garden, Lam Kam Road, Tai Po, Hong Kong SAR, China

[4] Forest Police Corps of Yunnan Public Security Department, Kunming, China

[5] Kunming Natural History Museum of Zoology, Kunming Institute of Zoology, Chinese Academy of Sciences, Kunming, China

[6] Department of Veterinary Medicine, University of Ibadan, Ibadan, Nigeria

[7] Southwest United Graduate School, Kunming, China

[*] These authors contributed equally to this work.

[#] **Corresponding authors:** Li Yu, Jing Yang Hu, Hua Rong Zhang

E-mail: yuli@ynu.edu.cn; hujingyang@ynu.edu.cn; hzhang@kfbg.org

Supplementary Information includes:

Figures S1-S3

Tables S1-S6

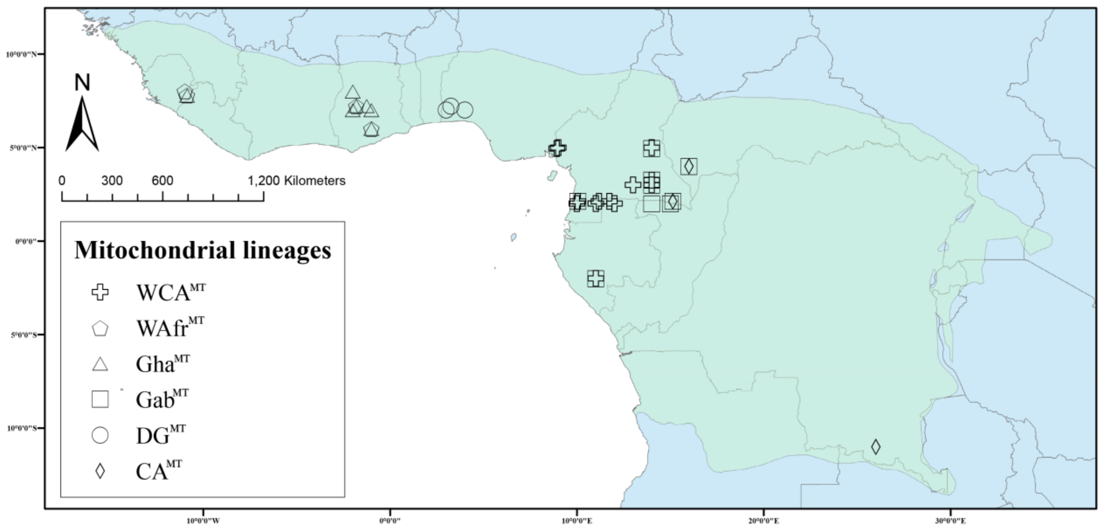

Figure S1. Sample information showing the overlapping distribution of the six mitochondrial lineages of white-bellied pangolin. The green range corresponds to the distribution area of white-bellied pangolins according to the IUCN Red List. Different symbols indicate different mitochondrial lineages of white-bellied pangolins.

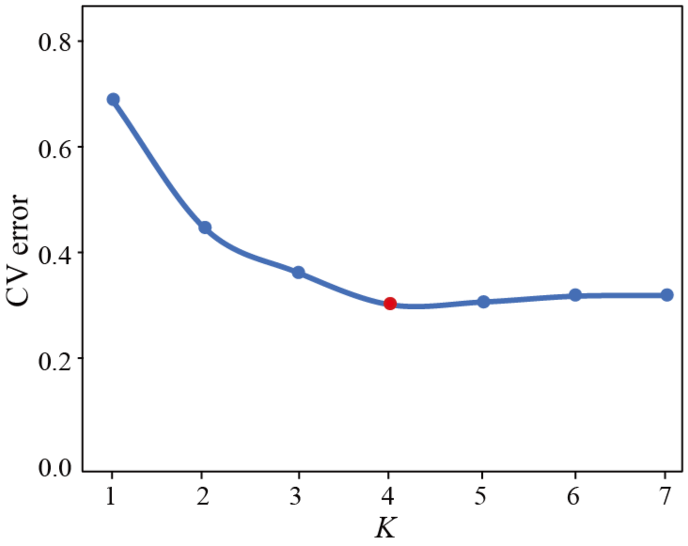

Figure S2. Optimal *K* value for Admixture analysis of white-bellied pangolin. Prediction error for cross-validation (CV error) of Admixture analysis of white-bellied pangolin, with the smallest error when *K*=4.

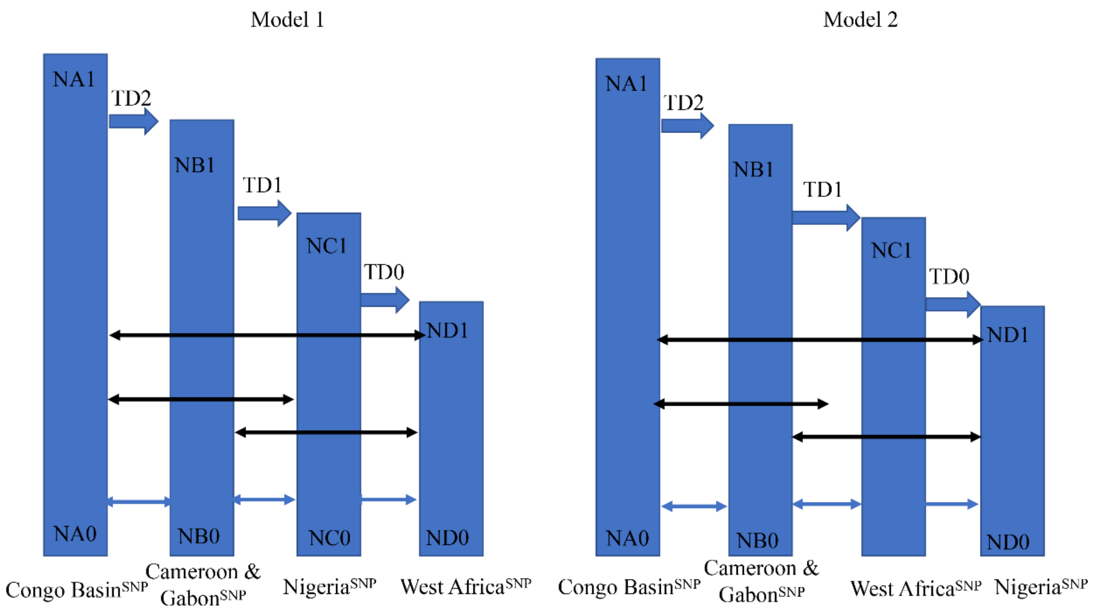

Figure S3. Demographic scenarios for white-bellied pangolin based on coalescent-based simulations.

Two ancestral dispersal models for the white-bellied pangolin were first developed. Model 1 represents the Nigeria^{SNP} lineage diverging earlier than the West Africa^{SNP} lineage, while Model 2 represents the Nigeria^{SNP} lineage diverging later than the West Africa^{SNP} lineage. Akaike information criterion (AIC) results indicate that Model 2 (AIC=1550617) is better than Model 1 (AIC=1717592).

Table S1. Newly obtained sequencing data in this study.

Number	Sample ID	Raw data (bp)	Raw data depth	Q20	Mapping (%)
1	PTR21	89,441,778,600	37.267	97.22	95.41%
2	PTR22	84,452,367,300	35.188	96.89	96.20%
3	PTR23	102,682,257,900	42.784	97.43	97.73%
4	PTR24	112,108,956,000	46.712	97.26	98.55%
5	PTR25	111,716,120,700	46.548	97.86	95.36%
6	PTR26	113,688,988,800	47.370	97.81	93.11%
7	PTR27	106,500,962,100	44.375	97.62	98.18%
8	PTR28	102,643,809,300	42.768	97.66	98.91%
9	PTR29	110,317,669,500	45.966	97.70	84.71%
10	PTR30	133,819,701,300	55.758	97.50	91.76%
11	PTR31	107,597,337,900	44.832	97.60	90.76%
12	PTR32	109,933,916,700	45.806	97.40	92.16%
13	PTR33	92,695,242,000	38.623	96.93	91.20%
14	PTR34	92,854,652,700	38.689	97.00	92.13%
15	PTR35	102,457,643,100	42.691	97.31	99.10%
16	PTR36	110,255,329,500	45.940	97.04	98.73%
17	PTR37	95,286,220,200	39.703	98.00	98.32%
18	PTR38	91,697,618,400	38.207	97.00	96.23%
19	PTR39	107,380,545,000	44.742	97.28	95.40%
20	PTR40	110,137,542,000	45.891	97.39	93.54%
21	PTR41	94,654,443,600	39.439	97.16	83.02%
22	PTR42	96,757,401,000	40.316	97.56	93.86%
23	PTR43	96,282,731,400	40.118	97.50	97.68%
24	PTR44	123,288,706,800	51.370	95.93	89.48%
25	PTR45	100,766,022,600	41.986	97.36	89.73%
26	PTR46	108,090,667,800	45.038	97.91	93.13%
27	PTR47	132,077,189,100	55.032	97.64	71.91%
28	PTR48	108,029,084,700	45.012	97.52	89.11%
29	PTR49	122,274,564,000	50.948	97.98	98.42%
30	PTR50	108,056,332,800	45.023	97.73	99.75%
31	PTR51	126,210,593,100	52.588	96.64	99.05%
32	PTR52	125,823,180,000	52.426	96.91	97.85%
33	PTR53	110,235,656,100	45.932	96.26	99.57%
34	PTR54	121,105,693,200	50.461	96.45	99.55%
35	PTR55	117,203,626,200	48.835	96.99	92.39%
36	PTR56	124,495,119,300	51.873	96.58	98.00%
37	PTR57	123,555,150,600	51.481	96.98	97.54%
38	PTR58	123,051,467,700	51.271	96.87	96.28%
39	PTR59	123,307,514,400	51.378	96.53	95.98%
40	PTR60	117,336,449,700	48.890	95.76	99.61%

41	PTR61	119,607,095,700	49.836	95.98	98.77%
42	PTR62	120,022,231,200	50.009	95.91	99.75%
43	PTR63	119,905,606,800	49.961	95.95	99.69%
44	PTR64	118,551,019,200	49.396	95.76	98.63%
45	PTR65	119,841,400,200	49.934	95.82	99.62%
46	PTR66	105,313,628,400	43.881	95.41	99.44%
47	PTR67	119,747,700,000	49.895	95.89	99.31%
48	PTR68	120,999,464,700	50.416	96.12	97.64%
49	PTR69	102,080,605,500	42.534	95.75	99.36%
50	PTR70	109,019,022,000	45.425	96.29	98.96%
51	PTR71	101,331,301,500	42.221	95.80	96.22%
52	PTR72	109,989,759,900	45.829	96.07	99.48%
53	PTR73	110,369,178,900	45.987	96.09	99.74%
54	PTR74	109,163,742,600	45.485	96.54	76.05%
55	PTR75	123,923,620,200	51.635	96.74	98.83%
56	PTR76	122,873,077,500	51.197	96.98	97.94%
57	PTR77	119,781,340,200	49.909	96.54	98.15%
58	PTR78	124,366,822,800	51.820	97.15	99.25%
59	PTR79	124,640,040,600	51.933	97.19	99.21%
60	PTR80	113,053,813,500	47.106	96.96	97.12%
61	PTR81	121,653,057,900	50.689	96.85	96.94%
62	PTR82	120,699,638,100	50.292	97.39	97.64%
63	PTR83	119,209,115,100	49.670	96.61	95.81%
64	PTR84	119,554,137,300	49.814	97.08	95.37%
65	PTR85	103,544,296,800	43.143	96.93	97.55%
66	PTR86	120,117,652,800	50.049	96.96	99.19%
67	PTR87	117,115,004,700	48.798	96.87	99.23%
68	PTR88	75,905,661,900	31.627	97.14	90.51%
69	PTR89	105,415,404,600	43.923	95.88	99.16%
70	PTR90	120,099,658,200	50.042	96.24	99.22%
71	PTR91	116,886,296,100	48.703	96.27	99.63%
72	PTR92	104,644,374,900	43.602	96.21	99.70%
73	PTR93	97,390,281,600	40.579	95.68	99.91%
74	PTR94	119,778,273,600	49.908	96.17	98.57%
75	PTR95	120,418,007,100	50.174	96.03	99.45%
76	PTR96	120,577,630,800	50.241	96.45	99.35%
77	PTR97	119,597,904,000	49.832	97.16	99.54%
78	PTR98	112,730,560,500	46.971	96.92	99.10%
79	PTR99	120,706,492,800	50.294	96.49	99.41%
80	PTR100	120,885,302,100	50.369	96.00	97.16%
81	PTR101	109,328,189,400	45.553	96.37	99.47%
82	PTR102	119,717,678,100	49.882	96.71	98.53%
83	PTR103	113,049,618,300	47.104	96.94	97.79%
84	PTR104	121,964,431,200	50.819	96.84	97.63%

85	PTR105	124,279,063,200	51.783	96.95	94.57%
86	PTR106	124,521,501,000	51.884	96.29	99.45%
87	PTR107	111,195,972,600	46.332	96.57	99.32%
88	PTR108	120,562,568,100	50.234	97.01	92.49%
89	PTR109	117,097,282,800	48.791	96.78	96.47%
90	PTR110	91,824,588,600	38.260	96.13	98.76%
91	PTR111	120,766,378,500	50.319	96.90	87.40%
92	PTR112	119,179,531,500	49.658	96.82	99.11%
93	PTR113	124,518,846,600	51.883	96.73	99.26%
94	PTR114	79,965,051,900	33.319	96.17	98.71%
95	PTR115	102,871,081,200	42.863	95.66	99.29%
96	PTR116	90,882,690,900	37.868	95.78	97.85%
97	PTR117	120,986,531,100	50.411	96.78	84.90%
98	PTR118	120,214,179,600	50.089	96.63	99.56%
99	PTR119	120,665,995,800	50.277	97.18	99.29%
100	PTR120	117,373,912,200	48.906	96.50	98.75%

Table S2. Geographic origin information for 209 samples, including the 122 assigned confiscated samples using *OriGen* and 87 published individuals with known geographic origin information from Tinsman et al. 2023.

Number	ID	Data Sources	Geographic lineage	rep_pofz	predLong	predLat	highprob	region	SNP Lineage	MT Lineage
1	PTR01	Gu et al. 2023	Cameroon & Gabon	1	8.913	4.957	0.61	Ndian, Southwest Region, Cameroon	Cameroon & $Gabon^{SNP}$	WCA^{MT}
2	PTR02	Gu et al. 2023	Manyu,Camero on	1	8.913	4.957	1	Ndian, southwestern Cameroon	Cameroon & $Gabon^{SNP}$	WCA^{MT}
3	PTR03	Gu et al. 2023	Cameroon & Gabon	1	14	3.261	0.49	Haut-Nyong, southern Cameroon	Cameroon & $Gabon^{SNP}$	WCA^{MT}
4	PTR04	Gu et al. 2023	Manyu,Camero on	0.98	8.913	4.957	1	Ndian, southwestern Cameroon	Cameroon & $Gabon^{SNP}$	WCA^{MT}
5	PTR05	Gu et al. 2023	Manyu,Camero on	1	8.913	4.957	1	Ndian, southwestern Cameroon	Cameroon & $Gabon^{SNP}$	WCA^{MT}
6	PTR06	Gu et al. 2023	Cameroon & Gabon	1	14	3.261	0.56	Haut-Nyong, southern Cameroon	Cameroon & $Gabon^{SNP}$	WCA^{MT}
7	PTR07	Gu et al. 2023	Cameroon & Gabon	1	10.043	2.13	0.95	Machinda, Litoral, Equatorial Guinea	Cameroon & $Gabon^{SNP}$	WCA^{MT}
8	PTR08	Gu et al. 2023	Cameroon & Gabon	1	10.043	2.13	0.3	Machinda, Litoral, Equatorial Guinea	Cameroon & $Gabon^{SNP}$	WCA^{MT}
9	PTR09	Gu et al. 2023	Cameroon & Gabon	1	11.174	2.13	0.51	ebebiyin, Kie Ntem, Equatorial Guinea	Cameroon & $Gabon^{SNP}$	WCA^{MT}
10	PTR10	Gu et al. 2023	Cameroon & Gabon	1	11.739	2.13	0.88	Ntem, Wouleu-Ntem, Gabon	Cameroon & $Gabon^{SNP}$	WCA^{MT}
11	PTR11	Gu et al. 2023	Cameroon & Gabon	1	14	3.261	0.75	Haut-Nyong, southern Cameroon	Cameroon & $Gabon^{SNP}$	WCA^{MT}
12	PTR12	Gu et al. 2023	Cameroon & Gabon	1	10.043	2.13	0.99	Machinda, Litoral, Equatorial Guinea	Cameroon & $Gabon^{SNP}$	WCA^{MT}
13	PTR13	Gu et al. 2023	Cameroon & Gabon	1	14	3.261	0.49	Haut-Nyong, southern Cameroon	Cameroon & $Gabon^{SNP}$	WCA^{MT}

14	PTR14	Gu et al. 2023	Cameroon & Gabon	1	10.043	2.13	0.86	Machinda, Litoral, Equatorial Guinea	Cameroon & Gabon^{SNP}	WCA^{MT}
15	PTR15	Gu et al. 2023	Cameroon & Gabon	1	14	3.261	0.5	Haut-Nyong, southern Cameroon	Cameroon & Gabon^{SNP}	WCA^{MT}
16	PTR16	Gu et al. 2023	Cameroon & Gabon	1	10.043	2.13	0.42	Machinda, Litoral, Equatorial Guinea	Cameroon & Gabon^{SNP}	WCA^{MT}
17	PTR17	Gu et al. 2023	Cameroon & Gabon	1	11.739	2.13	0.42	Ntem, Wouleu-Ntem, Gabon	Cameroon & Gabon^{SNP}	WCA^{MT}
18	PTR18	Gu et al. 2023	Cameroon & Gabon	1	11.739	2.13	0.61	Ntem, Wouleu-Ntem, Gabon	Cameroon & Gabon^{SNP}	WCA^{MT}
19	PTR19	Gu et al. 2023	Cameroon & Gabon	1	10.043	2.13	0.71	Machinda, Litoral, Equatorial Guinea	Cameroon & Gabon^{SNP}	WCA^{MT}
20	PTR20	Gu et al. 2023	Cameroon & Gabon	1	10.043	2.13	0.33	Machinda, Litoral, Equatorial Guinea	Cameroon & Gabon^{SNP}	WCA^{MT}
21	PTR21	This study	Congo Basin	1	15.13	2.13	1	Boumba-et-Ngoko, southeastern Cameroon	Congo Basin^{SNP}	CA^{MT}
22	PTR22	This study	Congo Basin	1	15.13	2.13	0.99	Boumba-et-Ngoko, southeastern Cameroon	Congo Basin^{SNP}	CA^{MT}
23	PTR23	This study	Manyu,Cameroon	1	8.913	4.957	1	Ndian, southwestern Cameroon	Cameroon & Gabon^{SNP}	WCA^{MT}
24	PTR24	This study	Cameroon & Gabon	1	10.043	2.13	0.81	Machinda, Litoral, Equatorial Guinea	Cameroon & Gabon^{SNP}	WCA^{MT}
25	PTR25	This study	West Africa	1	-10.87	7.783	0.78	Kailahun, Eastern, Sierra Leone	West Africa^{SNP}	WAfr^{MT}
26	PTR26	This study	West Africa	1	-10.87	7.783	0.78	Kailahun, Eastern, Sierra Leone	West Africa^{SNP}	WAfr^{MT}
27	PTR27	This study	Nigeria	1	3.261	7.217	0.34	Abeokuta North, Ogun, Nigeria	Nigeria^{SNP}	DG^{MT}
28	PTR28	This study	Nigeria	1	3.261	7.217	0.4	Abeokuta North, Ogun, Nigeria	Nigeria^{SNP}	DG^{MT}
29	PTR29	This study	Congo Basin	1	15.13	2.13	0.97	Boumba-et-Ngoko, southeastern Cameroon	Congo Basin^{SNP}	Gab^{MT}
30	PTR30	This study	Congo Basin	1	15.13	2.13	1	Boumba-et-Ngoko, southeastern Cameroon	Congo Basin^{SNP}	Gab^{MT}

31	PTR31	This study	West Africa	1	-10.87	7.783	0.48	Kailahun, Eastern, Sierra Leone	West Africa^{SNP}	WAfr^{MT}
32	PTR32	This study	Congo Basin	1	15.13	2.13	1	Boumba-et-Ngoko, southeastern Cameroon	Congo Basin^{SNP}	Gab^{MT}
33	PTR33	This study	Congo Basin	1	15.13	2.13	0.98	Boumba-et-Ngoko, southeastern Cameroon	Congo Basin^{SNP}	Gab^{MT}
34	PTR34	This study	Congo Basin	1	15.13	2.13	0.95	Boumba-et-Ngoko, southeastern Cameroon	Congo Basin^{SNP}	Gab^{MT}
35	PTR35	This study	Congo Basin	1	15.13	2.13	0.99	Boumba-et-Ngoko, southeastern Cameroon	Congo Basin^{SNP}	Gab^{MT}
36	PTR36	This study	West Africa	1	-10.87	7.783	0.78	Kailahun, Eastern, Sierra Leone	West Africa^{SNP}	WAfr^{MT}
37	PTR37	This study	West Africa	1	-10.87	7.783	0.7	Kailahun, Eastern, Sierra Leone	West Africa^{SNP}	WAfr^{MT}
38	PTR38	This study	Nigeria	1	3.261	7.217	0.18	Abeokuta North, Ogun, Nigeria	Nigeria^{SNP}	DG^{MT}
39	PTR39	This study	West Africa	1	-10.87	7.783	0.61	Kailahun, Eastern, Sierra Leone	West Africa^{SNP}	WAfr^{MT}
40	PTR40	This study	Cameroon & Gabon	1	14	3.261	0.29	Haut-Nyong, southern Cameroon	Cameroon & Gabon^{SNP}	Gab^{MT}
41	PTR41	This study	Congo Basin	1	15.13	2.13	1	Boumba-et-Ngoko, southeastern Cameroon	Congo Basin^{SNP}	Gab^{MT}
42	PTR42	This study	Cameroon & Gabon	1	10.043	2.13	0.76	Machinda, Litoral, Equatorial Guinea	Cameroon & Gabon^{SNP}	Gab^{MT}
43	PTR43	This study	Nigeria	1	3.261	7.217	0.28	Abeokuta North, Ogun, Nigeria	Nigeria^{SNP}	DG^{MT}
44	PTR44	This study	Congo Basin	1	15.13	2.13	0.96	Boumba-et-Ngoko, southeastern Cameroon	Congo Basin^{SNP}	Gab^{MT}
45	PTR45	This study	Congo Basin	1	15.13	2.13	0.99	Boumba-et-Ngoko, southeastern Cameroon	Congo Basin^{SNP}	Gab^{MT}
46	PTR46	This study	Nigeria	1	3.261	7.217	0.3	Abeokuta North, Ogun, Nigeria	Nigeria^{SNP}	DG^{MT}
47	PTR47	This study	Nigeria	1	3.261	7.217	0.17	Abeokuta North, Ogun, Nigeria	Nigeria^{SNP}	DG^{MT}

48	PTR48	This study	Nigeria	1	3.261	7.217	0.22	Abeokuta North, Ogun, Nigeria	Nigeria^{SNP}	DG^{MT}
49	PTR49	This study	Nigeria	1	3.261	7.217	0.14	Abeokuta North, Ogun, Nigeria	Nigeria^{SNP}	DG^{MT}
50	PTR50	This study	Nigeria	1	3.261	7.217	0.35	Abeokuta North, Ogun, Nigeria	Nigeria^{SNP}	DG^{MT}
51	PTR51	This study	Nigeria	1	3.261	7.217	0.19	Abeokuta North, Ogun, Nigeria	Nigeria^{SNP}	DG^{MT}
52	PTR52	This study	Nigeria	1	3.261	7.217	0.18	Abeokuta North, Ogun, Nigeria	Nigeria^{SNP}	DG^{MT}
53	PTR53	This study	Nigeria	1	3.261	7.217	0.44	Abeokuta North, Ogun, Nigeria	Nigeria^{SNP}	DG^{MT}
54	PTR54	This study	Nigeria	1	3.261	7.217	0.12	Abeokuta North, Ogun, Nigeria	Nigeria^{SNP}	DG^{MT}
55	PTR55	This study	Manyu,Cameroon	1	8.913	4.957	1	Ndian, southwestern Cameroon	Cameroon & Gabon^{SNP}	WCA^{MT}
56	PTR56	This study	Nigeria	1	3.261	7.217	0.22	Abeokuta North, Ogun, Nigeria	Nigeria^{SNP}	DG^{MT}
57	PTR57	This study	Nigeria	1	3.261	7.217	0.11	Abeokuta North, Ogun, Nigeria	Nigeria^{SNP}	DG^{MT}
58	PTR58	This study	Nigeria	1	3.261	7.217	0.2	Abeokuta North, Ogun, Nigeria	Nigeria^{SNP}	DG^{MT}
59	PTR59	This study	Cameroon & Gabon	1	11.739	2.13	1	Ntem, Wouleu-Ntem, Gabon	Congo Basin^{SNP}	WCA^{MT}
60	PTR60	This study	West Africa	1	-1.826	7.217	0.5	Ofinso, Ashanti, Ghana	Congo Basin^{SNP}	WAfr^{MT}
61	PTR61	This study	West Africa	1	-1.826	7.217	0.51	Ofinso, Ashanti, Ghana	West Africa^{SNP}	WAfr^{MT}
62	PTR62	This study	West Africa	1	-10.87	7.783	0.74	Kailahun, Eastern, Sierra Leone	West Africa^{SNP}	WAfr^{MT}
63	PTR63	This study	West Africa	1	-1.826	7.217	0.51	Ofinso, Ashanti, Ghana	West Africa^{SNP}	WAfr^{MT}
64	PTR64	This study	West Africa	1	-10.87	7.783	0.79	Kailahun, Eastern, Sierra Leone	West Africa^{SNP}	WAfr^{MT}
65	PTR65	This study	West Africa	1	-10.87	7.783	0.67	Kailahun, Eastern, Sierra Leone	West Africa^{SNP}	WAfr^{MT}

66	PTR66	This study	West Africa	1	-10.87	7.783	0.75	Kailahun, Eastern, Sierra Leone	West Africa^{SNP}	WAfr^{MT}
67	PTR67	This study	West Africa	1	-1.826	7.217	0.44	Ofinso, Ashanti, Ghana	West Africa^{SNP}	WAfr^{MT}
68	PTR68	This study	West Africa	1	-10.87	7.783	0.75	Kailahun, Eastern, Sierra Leone	West Africa^{SNP}	WAfr^{MT}
69	PTR69	This study	West Africa	1	-10.87	7.783	0.78	Kailahun, Eastern, Sierra Leone	West Africa^{SNP}	WAfr^{MT}
70	PTR70	This study	West Africa	1	-1.826	7.217	0.48	Ofinso, Ashanti, Ghana	West Africa^{SNP}	WAfr^{MT}
71	PTR71	This study	West Africa	1	-10.87	7.783	0.74	Kailahun, Eastern, Sierra Leone	West Africa^{SNP}	WAfr^{MT}
72	PTR72	This study	West Africa	1	-10.87	7.783	0.83	Kailahun, Eastern, Sierra Leone	West Africa^{SNP}	WAfr^{MT}
73	PTR73	This study	West Africa	1	-10.87	7.783	0.79	Kailahun, Eastern, Sierra Leone	West Africa^{SNP}	WAfr^{MT}
74	PTR74	This study	West Africa	1	-1.826	7.217	0.43	Ofinso, Ashanti, Ghana	West Africa^{SNP}	Gha^{MT}
75	PTR75	This study	Nigeria	1	3.261	7.217	0.28	Abeokuta North, Ogun, Nigeria	Nigeria^{SNP}	DG^{MT}
76	PTR76	This study	Nigeria	1	3.261	7.217	0.17	Abeokuta North, Ogun, Nigeria	Nigeria^{SNP}	DG^{MT}
77	PTR77	This study	Cameroon & Gabon	1	10.043	2.13	0.37	Machinda, Litoral, Equatorial Guinea	Cameroon & Gabon^{SNP}	WCA^{MT}
78	PTR78	This study	Cameroon & Gabon	1	10.043	2.13	0.28	Machinda, Litoral, Equatorial Guinea	Cameroon & Gabon^{SNP}	WCA^{MT}
79	PTR79	This study	Nigeria	1	3.261	7.217	0.17	Abeokuta North, Ogun, Nigeria	Nigeria^{SNP}	DG^{MT}
80	PTR80	This study	Nigeria	1	3.261	7.217	0.14	Abeokuta North, Ogun, Nigeria	Nigeria^{SNP}	DG^{MT}
81	PTR81	This study	Nigeria	1	3.261	7.217	0.25	Abeokuta North, Ogun, Nigeria	Nigeria^{SNP}	DG^{MT}
82	PTR82	This study	Cameroon & Gabon	1	14	3.261	0.43	Haut-Nyong, southern Cameroon	Cameroon & Gabon^{SNP}	Gab^{MT}
83	PTR83	This study	Congo Basin	1	15.13	2.13	0.99	Boumba-et-Ngoko, southeastern Cameroon	Congo Basin^{SNP}	Gab^{MT}

84	PTR84	This study	Congo Basin	1	15.13	2.13	0.95	Boumba-et-Ngoko, southeastern Cameroon	Congo Basin^{SNP}	Gab^{MT}
85	PTR85	This study	Congo Basin	1	15.13	2.13	1	Boumba-et-Ngoko, southeastern Cameroon	Cameroon & Gabon^{SNP}	Gab^{MT}
86	PTR86	This study	Congo Basin	1	15.13	2.13	0.99	Boumba-et-Ngoko, southeastern Cameroon	Congo Basin^{SNP}	Gab^{MT}
87	PTR87	This study	Cameroon & Gabon	1	14	3.261	0.81	Haut-Nyong, southern Cameroon	Cameroon & Gabon^{SNP}	WCA^{MT}
88	PTR88	This study	Congo Basin	1	15.13	2.13	0.92	Boumba-et-Ngoko, southeastern Cameroon	Congo Basin^{SNP}	CA^{MT}
89	PTR89	This study	West Africa	1	-10.87	7.783	0.41	Kailahun, Eastern, Sierra Leone	West Africa^{SNP}	Gha^{MT}
90	PTR90	This study	West Africa	1	-1.826	7.217	0.68	Ofinso, Ashanti, Ghana	West Africa^{SNP}	WAfr^{MT}
91	PTR91	This study	West Africa	1	-1.826	7.217	0.35	Ofinso, Ashanti, Ghana	West Africa^{SNP}	WAfr^{MT}
92	PTR92	This study	West Africa	1	-1.826	7.217	0.82	Ofinso, Ashanti, Ghana	West Africa^{SNP}	WAfr^{MT}
93	PTR93	This study	West Africa	1	-1.261	7.217	0.01	Sekyere West, Ashanti, Ghana	West Africa^{SNP}	Gha^{MT}
94	PTR94	This study	West Africa	1	-1.826	7.217	0.48	Ofinso, Ashanti, Ghana	West Africa^{SNP}	WAfr^{MT}
95	PTR95	This study	West Africa	1	-10.87	7.783	0.53	Kailahun, Eastern, Sierra Leone	West Africa^{SNP}	WAfr^{MT}
96	PTR96	This study	West Africa	1	-10.87	7.783	0.79	Kailahun, Eastern, Sierra Leone	West Africa^{SNP}	WAfr^{MT}
97	PTR97	This study	West Africa	1	-1.826	7.217	0.35	Ofinso, Ashanti, Ghana	West Africa^{SNP}	WAfr^{MT}
98	PTR98	This study	West Africa	1	-1.826	7.217	0.86	Ofinso, Ashanti, Ghana	West Africa^{SNP}	WAfr^{MT}
99	PTR99	This study	West Africa	1	-1.826	7.217	0.82	Ofinso, Ashanti, Ghana	West Africa^{SNP}	WAfr^{MT}
100	PTR100	This study	West Africa	1	-1.826	7.217	0.67	Ofinso, Ashanti, Ghana	West Africa^{SNP}	WAfr^{MT}
101	PTR101	This study	Cameroon & Gabon	1	14	4.957	0.11	Lom-et-Djerem, southern Cameroon	Cameroon & Gabon^{SNP}	Gab^{MT}
102	PTR102	This study	Congo Basin	1	15.13	2.13	1	Boumba-et-Ngoko, southeastern Cameroon	Congo Basin^{SNP}	Gab^{MT}
103	PTR103	This study	Nigeria	1	3.261	7.217	0.11	Abeokuta North, Ogun, Nigeria	Nigeria^{SNP}	DG^{MT}

104	PTR104	This study	Nigeria	1	3.261	7.217	0.17	Abeokuta North, Ogun, Nigeria	Nigeria^{SNP}	DG^{MT}
105	PTR105	This study	Nigeria	1	3.261	7.217	0.17	Abeokuta North, Ogun, Nigeria	Nigeria^{SNP}	DG^{MT}
106	PTR106	This study	Cameroon & Gabon	1	8.913	4.957	0.09	Ndian, southwestern Cameroon	Cameroon & Gabon^{SNP}	WCA^{MT}
107	PTR107	This study	Congo Basin	1	15.13	2.13	0.98	Boumba-et-Ngoko, southeastern Cameroon	Congo Basin^{SNP}	Gab^{MT}
108	PTR108	This study	Congo Basin	1	15.13	2.13	0.99	Boumba-et-Ngoko, southeastern Cameroon	Congo Basin^{SNP}	Gab^{MT}
109	PTR109	This study	Cameroon & Gabon	1	11.739	2.13	0.68	Ntem, Wouleu-Ntem, Gabon	Cameroon & Gabon^{SNP}	WCA^{MT}
110	PTR110	This study	Cameroon & Gabon	1	14	3.261	0.89	Haut-Nyong, southern Cameroon	Cameroon & Gabon^{SNP}	WCA^{MT}
111	PTR111	This study	Nigeria	1	3.261	7.217	0.35	Abeokuta North, Ogun, Nigeria	Nigeria^{SNP}	DG^{MT}
112	PTR112	This study	Nigeria	1	3.261	7.217	0.34	Abeokuta North, Ogun, Nigeria	Nigeria^{SNP}	DG^{MT}
113	PTR113	This study	Manyu,Camero on	1	8.913	4.957	1	Ndian, southwestern Cameroon	Cameroon & Gabon^{SNP}	WCA^{MT}
114	PTR114	This study	Congo Basin	1	15.13	2.13	1	Boumba-et-Ngoko, southeastern Cameroon	Cameroon & Gabon^{SNP}	Gab^{MT}
115	PTR115	This study	Nigeria	1	3.261	7.217	0.32	Abeokuta North, Ogun, Nigeria	Nigeria^{SNP}	DG^{MT}
116	PTR116	This study	Nigeria	1	3.261	7.217	0.19	Abeokuta North, Ogun, Nigeria	Nigeria^{SNP}	DG^{MT}
117	PTR117	This study	Congo Basin	1	15.13	2.13	0.99	Boumba-et-Ngoko, southeastern Cameroon	Congo Basin^{SNP}	Gab^{MT}
118	PTR118	This study	Cameroon & Gabon	1	14	3.261	0.13	Haut-Nyong, southern Cameroon	Cameroon & Gabon^{SNP}	Gab^{MT}
119	PTR119	This study	Congo Basin	1	15.13	2.13	0.99	Boumba-et-Ngoko, southeastern Cameroon	Congo Basin^{SNP}	Gab^{MT}
120	PTR120	This study	Nigeria	1	3.261	7.217	0.31	Abeokuta North, Ogun, Nigeria	Nigeria^{SNP}	DG^{MT}

121	PTR121	Houck et al. 2023	Nigeria	1	3.261	7.217	0.3	Abeokuta North, Ogun, Nigeria	Nigeria^{SNP}	DG^{MT}
122	PTR122	Damas et al. 2022	Manyu,Cameroon	1	8.913	4.957	1	Ndian, southwestern Cameroon	West Africa^{SNP}	WCA^{MT}
123	SRR25996363	Tinsman et al. 2023	Cameroon & Gabon	/	10	2	/	Vallee du Ntem, southwestern Cameroon	Cameroon & Gabon^{SNP}	WCA^{MT}
124	SRR25996364	Tinsman et al. 2023	West Africa	/	-11	8	/	Eastern, Sierra Leone	West Africa^{SNP}	WAfr^{MT}
125	SRR25996365	Tinsman et al. 2023	West Africa	/	-11	8	/	Eastern, Sierra Leone	West Africa^{SNP}	WAfr^{MT}
126	SRR25996366	Tinsman et al. 2023	West Africa	/	-11	8	/	Eastern, Sierra Leone	West Africa^{SNP}	WAfr^{MT}
127	SRR25996367	Tinsman et al. 2023	West Africa	/	-11	8	/	Eastern, Sierra Leone	Cameroon & Gabon^{SNP}	WAfr^{MT}
128	SRR25996368	Tinsman et al. 2023	West Africa	/	-11	8	/	Eastern, Sierra Leone	Congo Basin^{SNP}	WAfr^{MT}
129	SRR25996369	Tinsman et al. 2023	Nigeria	/	3	7	/	Ogun, Nigeria	Nigeria^{SNP}	DG^{MT}
130	SRR25996371	Tinsman et al. 2023	Nigeria	/	3	7	/	Ogun, Nigeria	Nigeria^{SNP}	DG^{MT}
131	SRR25996372	Tinsman et al. 2023	Cameroon & Gabon	/	14	3	/	Haut-Nyong, southern Cameroon	Cameroon & Gabon^{SNP}	WCA^{MT}
132	SRR25996373	Tinsman et al. 2023	Nigeria	/	3	7	/	Ogun, Nigeria	Nigeria^{SNP}	DG^{MT}
133	SRR25996374	Tinsman et al. 2023	Nigeria	/	4	7	/	Ogun, Nigeria	Nigeria^{SNP}	DG^{MT}
134	SRR25996375	Tinsman et al. 2023	Nigeria	/	3	7	/	Ogun, Nigeria	Nigeria^{SNP}	DG^{MT}
135	SRR25996376	Tinsman et al. 2023	Nigeria	/	3	7	/	Ogun, Nigeria	Nigeria^{SNP}	DG^{MT}
136	SRR25996377	Tinsman et al. 2023	West Africa	/	-1	6	/	Eastern, Ghana	West Africa^{SNP}	Gha^{MT}
137	SRR25996378	Tinsman et al. 2023	West Africa	/	-2	8	/	Brong Ahafo, Ghana	West Africa^{SNP}	Gha^{MT}

138	SRR25996379	Tinsman et al. 2023	West Africa	/	-2	7	/	Ashanti, Ghana	West Africa[SNP]	Gha[MT]
139	SRR25996380	Tinsman et al. 2023	West Africa	/	-1	6	/	Eastern, Ghana	West Africa[SNP]	Gha[MT]
140	SRR25996381	Tinsman et al. 2023	West Africa	/	-2	8	/	Brong Ahafo, Ghana	West Africa[SNP]	Gha[MT]
141	SRR25996382	Tinsman et al. 2023	West Africa	/	-2	7	/	Ashanti, Ghana	West Africa[SNP]	Gha[MT]
142	SRR25996383	Tinsman et al. 2023	Cameroon & Gabon	/	14	3	/	Haut-Nyong, southern Cameroon	Congo Basin[SNP]	Gab[MT]
143	SRR25996384	Tinsman et al. 2023	West Africa	/	-2	7	/	Ashanti, Ghana	Congo Basin[SNP]	Gha[MT]
144	SRR25996385	Tinsman et al. 2023	West Africa	/	-2	7	/	Ashanti, Ghana	West Africa[SNP]	Gha[MT]
145	SRR25996386	Tinsman et al. 2023	West Africa	/	-1	6	/	Eastern, Ghana	West Africa[SNP]	Gha[MT]
146	SRR25996387	Tinsman et al. 2023	West Africa	/	-2	7	/	Ashanti, Ghana	Congo Basin[SNP]	Gha[MT]
147	SRR25996388	Tinsman et al. 2023	West Africa	/	-1	7	/	Ashanti, Ghana	West Africa[SNP]	Gha[MT]
148	SRR25996389	Tinsman et al. 2023	West Africa	/	-2	7	/	Ashanti, Ghana	West Africa[SNP]	Gha[MT]
149	SRR25996390	Tinsman et al. 2023	West Africa	/	-2	8	/	Brong Ahafo, Ghana	West Africa[SNP]	Gha[MT]
150	SRR25996391	Tinsman et al. 2023	West Africa	/	-1	6	/	Eastern, Ghana	West Africa[SNP]	Gha[MT]
151	SRR25996392	Tinsman et al. 2023	West Africa	/	-1	6	/	Eastern, Ghana	West Africa[SNP]	WAfr[MT]
152	SRR25996393	Tinsman et al. 2023	West Africa	/	-2	8	/	Brong Ahafo, Ghana	West Africa[SNP]	Gha[MT]
153	SRR25996394	Tinsman et al. 2023	Manyu,Cameroon	/	9	5	/	Manyu, southwestern Cameroon	Cameroon & Gabon[SNP]	WCA[MT]
154	SRR25996395	Tinsman et al. 2023	Cameroon & Gabon	/	11	-2	/	Moakabe, Gabon	Cameroon & Gabon[SNP]	Gab[MT]

155	SRR2599 6396	Tinsman et al. 2023	Cameroon & Gabon	/	11	-2	/	Libreville, Gabon	Cameroon & Gabon^{SNP}	WCA^{MT}
156	SRR2599 6397	Tinsman et al. 2023	Cameroon & Gabon	/	11	2	/	Bitam, Gabon	Cameroon & Gabon^{SNP}	WCA^{MT}
157	SRR2599 6398	Tinsman et al. 2023	Cameroon & Gabon	/	11	2	/	Bitam, Gabon	Cameroon & Gabon^{SNP}	WCA^{MT}
158	SRR2599 6399	Tinsman et al. 2023	Congo Basin	/	26	-11	/	Katanga, Democratic Republic of the Congo	Congo Basin^{SNP}	CA^{MT}
159	SRR2599 6400	Tinsman et al. 2023	Congo Basin	/	14	2	/	Republic of Congo	Congo Basin^{SNP}	Gab^{MT}
160	SRR2599 6401	Tinsman et al. 2023	Congo Basin	/	14	2	/	Republic of Congo	Congo Basin^{SNP}	Gab^{MT}
161	SRR2599 6402	Tinsman et al. 2023	Congo Basin	/	15	2	/	Republic of Congo	Congo Basin^{SNP}	Gab^{MT}
162	SRR2599 6403	Tinsman et al. 2023	Congo Basin	/	15	2	/	Republic of Congo	West Africa^{SNP}	Gab^{MT}
163	SRR2599 6404	Tinsman et al. 2023	Congo Basin	/	15	2	/	Republic of Congo	West Africa^{SNP}	Gab^{MT}
164	SRR2599 6405	Tinsman et al. 2023	Manyu,Camero on	/	9	5	/	Manyu, southwestern Cameroon	West Africa^{SNP}	WCA^{MT}
165	SRR2599 6406	Tinsman et al. 2023	Congo Basin	/	16	4	/	Central African Republic	West Africa^{SNP}	CA^{MT}
166	SRR2599 6407	Tinsman et al. 2023	Congo Basin	/	16	4	/	Central African Republic	West Africa^{SNP}	Gab^{MT}
167	SRR2599 6408	Tinsman et al. 2023	Cameroon & Gabon	/	14	5	/	Lom Djerem, eastern Cameroon	Cameroon & Gabon^{SNP}	WCA^{MT}
168	SRR2599 6409	Tinsman et al. 2023	Cameroon & Gabon	/	14	5	/	Lom Djerem, eastern Cameroon	Cameroon & Gabon^{SNP}	WCA^{MT}
169	SRR2599 6410	Tinsman et al. 2023	Cameroon & Gabon	/	14	5	/	Lom Djerem, eastern Cameroon	Cameroon & Gabon^{SNP}	WCA^{MT}
170	SRR2599 6411	Tinsman et al. 2023	Cameroon & Gabon	/	14	5	/	Lom Djerem, eastern Cameroon	Cameroon & Gabon^{SNP}	WCA^{MT}
171	SRR2599 6412	Tinsman et al. 2023	Cameroon & Gabon	/	14	5	/	Lom Djerem, eastern Cameroon	Cameroon & Gabon^{SNP}	WCA^{MT}

172	SRR25996413	Tinsman et al. 2023	Cameroon & Gabon	/	12	2	/	Mvila, southern Cameroon	Cameroon & Gabon[SNP]	WCA[MT]
173	SRR25996414	Tinsman et al. 2023	Cameroon & Gabon	/	12	2	/	Mvila, southern Cameroon	Cameroon & Gabon[SNP]	WCA[MT]
174	SRR25996415	Tinsman et al. 2023	Cameroon & Gabon	/	12	2	/	Haut-Nyong, southern Cameroon	Cameroon & Gabon[SNP]	WCA[MT]
175	SRR25996416	Tinsman et al. 2023	Cameroon & Gabon	/	12	2	/	Mvila, southern Cameroon	Cameroon & Gabon[SNP]	WCA[MT]
176	SRR25996417	Tinsman et al. 2023	Cameroon & Gabon	/	12	2	/	Mvila, southern Cameroon	Cameroon & Gabon[SNP]	WCA[MT]
177	SRR25996418	Tinsman et al. 2023	Cameroon & Gabon	/	12	2	/	Mvila, southern Cameroon	Cameroon & Gabon[SNP]	WCA[MT]
178	SRR25996419	Tinsman et al. 2023	Cameroon & Gabon	/	12	2	/	Mvila, southern Cameroon	Cameroon & Gabon[SNP]	WCA[MT]
179	SRR25996420	Tinsman et al. 2023	Cameroon & Gabon	/	13	3	/	Dja Lobo, southern Cameroon	Cameroon & Gabon[SNP]	WCA[MT]
180	SRR25996421	Tinsman et al. 2023	Cameroon & Gabon	/	14	3	/	Haut-Nyong, southern Cameroon	Cameroon & Gabon[SNP]	WCA[MT]
181	SRR25996422	Tinsman et al. 2023	Cameroon & Gabon	/	14	3	/	Haut-Nyong, southern Cameroon	Cameroon & Gabon[SNP]	WCA[MT]
182	SRR25996423	Tinsman et al. 2023	Cameroon & Gabon	/	14	3	/	Haut-Nyong, southern Cameroon	Cameroon & Gabon[SNP]	WCA[MT]
183	SRR25996424	Tinsman et al. 2023	Cameroon & Gabon	/	14	3	/	Haut-Nyong, southern Cameroon	Cameroon & Gabon[SNP]	WCA[MT]
184	SRR25996425	Tinsman et al. 2023	Cameroon & Gabon	/	10	2	/	Vallee du Ntem, southwestern Cameroon	Cameroon & Gabon[SNP]	WCA[MT]
185	SRR25996426	Tinsman et al. 2023	Cameroon & Gabon	/	14	3	/	Haut-Nyong, southern Cameroon	Cameroon & Gabon[SNP]	WCA[MT]
186	SRR25996427	Tinsman et al. 2023	Cameroon & Gabon	/	14	3	/	Haut-Nyong, southern Cameroon	Cameroon & Gabon[SNP]	WCA[MT]
187	SRR25996428	Tinsman et al. 2023	Cameroon & Gabon	/	14	3	/	Haut-Nyong, southern Cameroon	Cameroon & Gabon[SNP]	WCA[MT]
188	SRR25996429	Tinsman et al. 2023	Cameroon & Gabon	/	14	3	/	Haut-Nyong, southern Cameroon	Cameroon & Gabon[SNP]	WCA[MT]

189	SRR25996430	Tinsman et al. 2023	Cameroon & Gabon	/	12	2	/	Mvila, southern Cameroon	Cameroon & Gabon^{SNP}	WCA^{MT}
190	SRR25996431	Tinsman et al. 2023	Manyu,Cameroon	/	9	5	/	Manyu, southwestern Cameroon	Cameroon & Gabon^{SNP}	WCA^{MT}
191	SRR25996432	Tinsman et al. 2023	Manyu,Cameroon	/	9	5	/	Manyu, southwestern Cameroon	Cameroon & Gabon^{SNP}	WCA^{MT}
192	SRR25996433	Tinsman et al. 2023	Manyu,Cameroon	/	9	5	/	Manyu, southwestern Cameroon	Cameroon & Gabon^{SNP}	WCA^{MT}
193	SRR25996434	Tinsman et al. 2023	Cameroon & Gabon	/	10	2	/	Vallee du Ntem, southwestern Cameroon	Cameroon & Gabon^{SNP}	WCA^{MT}
194	SRR25996435	Tinsman et al. 2023	Cameroon & Gabon	/	10	2	/	Vallee du Ntem, southwestern Cameroon	Cameroon & Gabon^{SNP}	WCA^{MT}
195	SRR25996436	Tinsman et al. 2023	Cameroon & Gabon	/	14	3	/	Haut-Nyong, southern Cameroon	Cameroon & Gabon^{SNP}	WCA^{MT}
196	SRR25996437	Tinsman et al. 2023	Manyu,Cameroon	/	9	5	/	Manyu, southwestern Cameroon	Cameroon & Gabon^{SNP}	WCA^{MT}
197	SRR25996438	Tinsman et al. 2023	Cameroon & Gabon	/	10	2	/	Vallee du Ntem, southwestern Cameroon	Cameroon & Gabon^{SNP}	WCA^{MT}
198	SRR25996439	Tinsman et al. 2023	Cameroon & Gabon	/	10	2	/	Haut-Nyong, southern Cameroon	Cameroon & Gabon^{SNP}	WCA^{MT}
199	SRR25996440	Tinsman et al. 2023	Cameroon & Gabon	/	10	2	/	Vallee du Ntem, southwestern Cameroon	Cameroon & Gabon^{SNP}	WCA^{MT}
200	SRR25996441	Tinsman et al. 2023	Cameroon & Gabon	/	14	3	/	Haut-Nyong, southern Cameroon	Cameroon & Gabon^{SNP}	Gab^{MT}
201	SRR25996442	Tinsman et al. 2023	Cameroon & Gabon	/	13	3	/	Dja Lobo, southern Cameroon	Cameroon & Gabon^{SNP}	WCA^{MT}
202	SRR25996443	Tinsman et al. 2023	Cameroon & Gabon	/	10	2	/	Haut-Nyong, southern Cameroon	Cameroon & Gabon^{SNP}	WCA^{MT}
203	SRR25996444	Tinsman et al. 2023	Cameroon & Gabon	/	14	3	/	Vallee du Ntem, southwestern Cameroon	Cameroon & Gabon^{SNP}	WCA^{MT}
204	SRR25996445	Tinsman et al. 2023	Manyu,Cameroon	/	9	5	/	Manyu, southwestern Cameroon	Cameroon & Gabon^{SNP}	WCA^{MT}
205	SRR25996446	Tinsman et al. 2023	Cameroon & Gabon	/	14	3	/	Haut-Nyong, southern Cameroon	West Africa^{SNP}	WCA^{MT}

206	SRR2599 6447	Tinsman et al. 2023	Cameroon & Gabon	/	12	2	/	Mvila, southern Cameroon	Cameroon & Gabon[SNP]	WCA[MT]
207	SRR2599 6448	Tinsman et al. 2023	Manyu,Camero on	/	9	5	/	Manyu, southwestern Cameroon	Cameroon & Gabon[SNP]	WCA[MT]
208	SRR2599 6449	Tinsman et al. 2023	Cameroon & Gabon	/	12	2	/	Mvila, southern Cameroon	Cameroon & Gabon[SNP]	WCA[MT]
209	SRR2599 6450	Tinsman et al. 2023	Cameroon & Gabon	/	10	2	/	Vallee du Ntem, southwestern Cameroon	Cameroon & Gabon[SNP]	WCA[MT]

Table S3. F_{ST} values among the four white-bellied pangolin populations based on autosome SNPs.

	West Africa^{SNP}	Nigeria^{SNP}	Cameroon & Gabon^{SNP}	Congo Basin^{SNP}
West Africa^{SNP}	-	-	-	-
Nigeria^{SNP}	0.608	-	-	-
Cameroon & Gabon^{SNP}	0.521	0.499	-	-
Congo Basin^{SNP}	0.667	0.672	0.376	-

Table S4. Divergence time estimates for white-bellied pangolin based on single-copy orthologous sequences.

Split	Divergence time (Ma)	95% HPD (Ma)
Carnivora - Pholidota	79.18	67.67-87.75
Feliformia - Caniformia	51.97	37.82-66.07
Asian - African pangolins	35.72	30.35-43.72
Phataginus - *Smutsia*	18.96	8.87-29.56
Congo Basin^{SNP} - Cameroon & Gabon^{SNP}, West Africa^{SNP} and Nigeria^{SNP}	2.86	1.12-5.26
Cameroon & Gabon^{SNP} - West Africa^{SNP} and Nigeria^{SNP}	1.99	0.74-3.68
West Africa^{SNP} - Nigeria^{SNP}	1.11	0.33-2.15

Table S5. Divergence time estimates for white-bellied pangolin based on mitochondrial genes.

Split	Divergence time (Ma)	95% HPD (Ma)
Carnivora - Pholidota	75.67	66.31-87.14
Feliformia - Caniformia	50.67	36.99-65.38
Asian - African pangolins	38.22	31.11-45.07
Phataginus - *Smutsia*	23.1	13.2-33.91
Phataginus	10.7	7.32-13.13
CA^{MT} - Gab^{MT}	3.94	1.47-6.64
WCA^{MT} - $WAfr^{MT}$, Gha^{MT} and DG^{MT}	3.04	1.19-5.26
$WAfr^{MT}$ - Gha^{MT} and DG^{MT}	1.73	0.53-3.22
Gha^{MT} - DG^{MT}	1.51	0.36-2.94

Table S6. Gene flow estimation between the four genome-data genetic lineages of the white-bellied pangolin based on the relationships revealed in the phylogenetic trees. |Z-score| values >= 3 are shown in bold.

P1	P2	P3	D-statistic	Z-score	p-value	f4-ratio	BBAA	ABBA	BABA
West Africa^{SNP}	**Nigeria^{SNP}**	**Congo Basin^{SNP}**	0.02	**4.77**[*]	1.80417e-06	0.01	644333	69249.7	66350.3
Nigeria^{SNP}	**Cameroon & Gabon^{SNP}**	**Congo Basin^{SNP}**	0.25	**79.44**[**]	2.3e-16	0.29	370249	210773	127084
West Africa^{SNP}	**Nigeria^{SNP}**	**Cameroon & Gabon^{SNP}**	0.13	**25.24**[**]	2.3e-16	0.08	435523	103605	80014
West Africa^{SNP}	**Cameroon & Gabon^{SNP}**	**Congo Basin^{SNP}**	0.25	**68.42**[**]	2.3e-16	0.30	352740	216856	130268

[*] Significant at |z| >= 3.

[**] Significant at |z| >= 5.
